# High‐Resolution Genomic Resources for Trait Mapping and Precision Breeding for Adzuki Bean (*Vigna angularis*)

**DOI:** 10.1002/advs.202507157

**Published:** 2025-11-19

**Authors:** Liangliang Hu, Xiaolei Wang, Yazhou Zhang, Vanika Garg, Xingxing Yuan, Renfeng Xue, Hongmei Zhou, Yanhua Chen, Weili Hu, Rutwik Barmukh, Rong Cao, Tianxiao Chen, Qiannan Song, Dan Gao, Gaoling Luo, Xu Zhu, Xin Jing, Suhua Wang, Caiju Li, Yunshan Wei, Weide Ge, Xin Chen, Lixia Wang, Rajeev K Varshney, Chengzhi Jiao, Xuzhen Cheng, Honglin Chen

**Affiliations:** ^1^ State Key Laboratory of Crop Gene Resources and Breeding/Institute of Crop Sciences, Chinese Academy of Agricultural Sciences Beijing 100081 China; ^2^ Chifeng Academy of Agricultural and Animal Husbandry Sciences Chifeng 024031 China; ^3^ Smartgenomics Technology Institute Tianjin 301799 China; ^4^ Centre for Crop and Food Innovation WA State Agricultural Biotechnology Centre Food Futures Institute Murdoch University Murdoch WA 6150 Australia; ^5^ Institute of Industrial Crops Jiangsu Academy of Agricultural Sciences Nanjing 210014 China; ^6^ Crop Research Institute Liaoning Academy of Agricultural Sciences Shenyang 110161 China; ^7^ Baoding Academy of Agricultural Sciences Baoding 071051 China; ^8^ Vegetable Research Institute Guangxi Academy of Agricultural Sciences Nanning 530007 China; ^9^ Nanyang Academy of Agricultural Sciences Nanyang 473083 China

**Keywords:** adzuki bean, domestication, genetic improvement, genomics, precision breeding

## Abstract

Adzuki bean (*Vigna angularis*), a globally important legume crop, faces breeding bottlenecks due to limited genomic resources and an insufficient understanding of its genetic basis for key traits, which constrains the efficient utilization of its genetic diversity in breeding programs. To address this, a high‐quality genome assembly is developed for the elite cultivar ZH20 and a comprehensive genetic variation map is constructed by resequencing of 546 diverse adzuki bean accessions. Genomic and phenotypic analyses of this diversity panel reveal distinct population structures and identify genomic variations underlying key agronomic traits, including seed coat color, size, shape, and flowering time, linked to adaptation and selection. This analysis pinpointed 251 loci significantly associated with eight key agronomic traits, highlighting promising candidate genes, such as *ANKRD50* and *NAC73* for seed morphology, *ANR1* for flavonoid content, and *NPF5.4* for flowering time. Furthermore, comparative genomics provides insights into domestication processes. These datasets are integrated to develop *AdzukiBeanAtlas* (https://www.cgris.net/AdzukiBeanAtlas), a versatile toolkit to facilitate breeding strategies. These resources provide a valuable foundation for understanding adzuki bean diversity, while *AdzukiBeanAtlas* serves as a user‐friendly, cross‐platform tool for molecular marker development, helping to accelerate future breeding programs.

## Graphical abstract

This study establishes crucial genomic resources for adzuki bean, including a reference genome, variation map, and the *AdzukiBeanAtlas* toolkit. We identify key candidate genes (*ANKRD50*, *NAC73*, *ANR1*, *NPF5.4*) for important agronomic traits through Genome‐Wide Association Studies (GWAS). toolkit facilitates breeding design by leveraging these findings. These foundational resources enhance understanding of adzuki bean diversity and adaptation, significantly accelerating the development of improved cultivars.

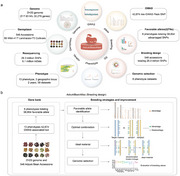

## 1 Introduction

Adzuki bean (*Vigna angularis*) is an economically significant legume widely cultivated across Asia for its nutritional value. It is a rich source of protein, starch, minerals, and micronutrients^[^ ^1^, ^2^ ^]^ as well as bioactive compounds such as flavonoids, phenolic acids, and saponins, which are linked to its traditional use in medicine. These compounds have led to traditional uses in Chinese medicine for purported diuretic and hypoglycemic properties.^[^ ^3^ ^]^ Beyond its nutritional and medicinal value, adzuki bean possesses inherent resistance to biotic and abiotic stresses, such as drought and salt tolerance, and can thrive in low‐fertility soils. These characteristics make it a valuable climate‐resilient crop, well‐suited to addressing food security challenges, particularly in Asia.^[^ ^4^ ^]^

Adzuki bean is believed to have originated in China, likely domesticated from its wild progenitor, *V. angularis* var. *nipponensis*.^[^ ^5^, ^6^ ^]^ Archaeological evidence indicates domestication may have begun as early as 12000 years ago.^[^ ^7^, ^8^ ^]^ Morphological and molecular data suggest the possibility of multiple independent domestication events across East Asia, including China, Japan, and Korea.^[^ ^9^ ^]^ China remains the world's largest producer and holds a significant germplasm collection, exceeding 5000 accessions. However, modern adzuki bean cultivars have predominantly been selected from landraces, resulting in a relatively narrow genetic base within the cultivated gene pool.^[^ ^10^, ^11^ ^]^ This narrow genetic diversity, coupled with underutilized genomic resources, has substantially constrained the pace of modern breeding progress.

Genomic selection (GS) presents a powerful strategy to accelerate this process. GS utilizes genome‐wide markers to predict the genomic estimated breeding values (GEBVs) of individuals, thereby reducing reliance solely on extensive phenotypic evaluation.^[^ ^12^, ^13^ ^]^ By training statistical models on a population with both genotypic and phenotypic data, GS enables the prediction of performance in selection candidates possessing only genotypic data.^[^ ^14^ ^]^ This predictive capability allows breeders to identify and select superior individuals at earlier developmental stages, before extensive and costly field trials, thereby significantly shortening the breeding cycle and enhancing the rate of genetic gain.^[^ ^15^ ^]^ Unlike traditional marker‐assisted selection (MAS) targeting a few major genes, GS excels at improving complex, polygenic traits controlled by numerous small‐effect loci. This capability is particularly relevant for adzuki bean, where many critical agronomic traits exhibit such a genetic architecture. By utilizing genome‐wide marker information, GS offers a more holistic approach to predict breeding values. Its proven success in enhancing breeding efficiency for complex traits in other major crops, including legumes like soybean,^[^ ^16^ ^]^ and cereals,^[^ ^17^, ^18^, ^19^ ^]^ strongly supports the potential of GS to significantly advance adzuki bean improvement. However, realizing the full potential of GS and other genomics‐assisted breeding approaches depends on the availability of comprehensive genomic resources.

While previous draft genomes provided valuable information,^[^ ^20^, ^21^, ^22^ ^]^ they suffered from fragmentation. Although a recent high‐quality reference genome represented a significant advance,^[^ ^11^ ^]^ further improvements in contiguity and completeness were needed. This study fills that gap by presenting a *de novo*, chromosome‐scale, and high‐quality reference genome assembly for the elite cultivar ZH20, selected based on its widespread use and high yield. Our assembly, integrating Illumina short reads, PacBio long reads, and Hi‐C data, achieves a contig N50 of 28.76 Mb, a substantial improvement over existing assemblies. Using this new assembly as a reference, we constructed a comprehensive genomic variation map by resequencing a distinct and diverse panel of 546 adzuki bean accessions, including wild types, landraces, and modern cultivars. We conducted genome‐wide association studies (GWAS) and population genomic analyses to dissect genetic diversity, population structure, domestication history, and selection signatures underlying phenotypic variation. To maximize the utility of these resources, we developed *AdzukiBeanAtlas*, a publicly accessible database and analysis platform. To our knowledge, *AdzukiBeanAtlas* is one of the most comprehensive platforms for legume breeding, integrating genomic and phenotypic data with a user‐friendly interface to support genomic selection, marker discovery, and the breeding of climate‐resilient adzuki bean cultivars. Ultimately, this work provides a vital foundation to accelerate adzuki bean breeding and contribute to global food security.

## 2 Results

### 2.1 Genome Assembly and Identification of Copy Number Variation

**Figure 1:**
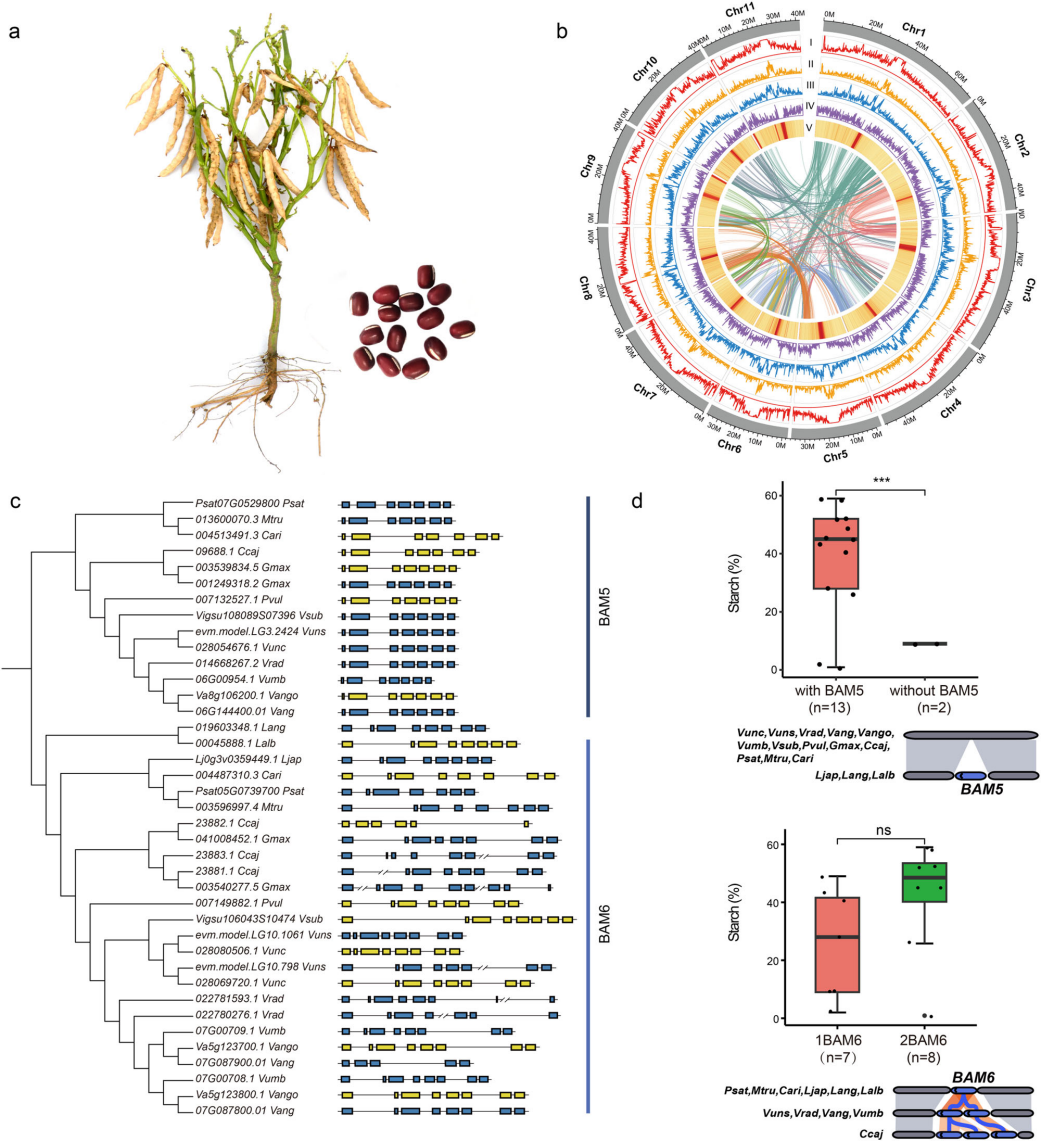
Genome assembly, annotation, and copy number variation analysis of the adzuki bean cultivar "ZH20". a) A mature “ZH20” plant. b) Genomic features of the “ZH20” reference genome. The Circos plot displays 11 pseudochromosomes (in Mb). From the outer to inner tracks: I) Density of transposable elements (TEs), II) Density of LTR‐Copia elements, III) Density of LTR‐Gypsy elements, IV) Gene density, and V) GC content. Links in the center depict syntenic relationships between chromosomes. Densities were calculated in 100 kb sliding windows with a 10 kb step. c) Genomic organization of the beta‐amylase genes *BAM5* and *BAM6*. *BAM5* exhibits presence/absence variation (PAV), while *BAM6* shows copy number variation. Blue and yellow boxes represent exons on the positive and negative strands, respectively. d) Association between gene variation and starch content. The presence of *BAM5* is significantly correlated with higher starch content (**P < 0.001, Student's *t*‐test). No significant correlation was found between *BAM6* copy number and starch content (ns, *P* > 0.05, Student's *t*‐test). The multiple copies of *BAM6* are arranged in tandem. Species abbreviations: *Psat*, *Pisum sativum*; *Mtru*, *Medicago truncatula*; *Cari*, *Cicer arietinum*; *Ljap*, *Lotus japonicus*; *Lang*, *Lupinus angustifolius*; *Lalb*, *Lupinus albus*; *Vunc*, *Vigna unguiculata*; *Vuns*, *Vigna unguiculata*; *Vang*, *Vigna angularis*; *Vmun*, *Vigna mungo*; *Vsub*, *Vigna subterranea*; *Ccaj*, *Cajanus cajan*; *Vumb*, *Vigna umbellata*; *Gmax*, *Glycine max*; *Pvul*, *Phaseolus vulgaris*; *Vrad*, *Vigna radiata*.

To establish a foundational genomic resource for adzuki bean, we generated a *de* *novo* reference genome for the elite cultivar “ZH20” (**Figure** **1**a). We generated 41.36 Gb of PacBio HiFi data (76.17x coverage) and 72.17 Gb of Hi‐C data (132.91x coverage) (Figure S1 and Table S1, Supporting Information). This yielded a chromosome‐scale assembly of 517.93 Mb, with 99.94% of the sequence anchored to 11 chromosomes (Figure S2, Supporting Information). The assembly is highly contiguous, with a contig N50 of 28.76 Mb, and exhibits exceptional completeness, with a long terminal repeat (LTR) assembly index (LAI) of 16.48% and a 99.1% BUSCO score using the embryophyta database (Figure 1b; Figures S3,S4; Tables S2,S3, Supporting Information). These metrics represent significant improvements in assembly quality and contiguity over previously published adzuki bean genomes.^[^ ^20^, ^21^, ^22^ ^]^ Notably, our assembly contains more completely annotated centromeric regions (totaling 25.8 Mb) than the Jingnong6 assembly (totaling 19.2 Mb) (Figures S5,S6 and Table S4, Supporting Information). Furthermore, our assembly successfully identified telomeric repeat sequences at the ends of all 11 chromosomes, indicating a high level of structural completeness (Table S5, Supporting Information).

Repetitive sequences accounted for 48.27% of the “ZH20” genome, with Gypsy (22.78%) and Copia (11.80%) being the most abundant transposable elements (Table S6, Supporting Information). Using a combination of *de novo* and homology‐based approaches, we predicted and annotated 30278 protein‐coding genes (Tables S7–S8, Supporting Information), with an average CDS length of 1125.01 bp and an average gene length of 3911.56 bp, and comprising an average of 4.90 exons per gene. Of these, 30082 genes (94.94%) were assigned a putative function based on comparisons with publicly available databases (Table S9, Supporting Information). Additionally, non‐coding RNA (ncRNA) annotation revealed rRNA as the most abundant type, accounting for 0.22% of the genome (Table S10, Supporting Information). In summary, the “ZH20” genome exhibits greater continuity and completeness than previously published *V. angularis* genomes, providing an excellent foundation for further adzuki bean research.

To investigate the evolutionary relationships, we performed gene family ortholog analysis using 16 legume species and *Arabidopsis thaliana*.^[^ ^18^ ^]^ The phylogenetic analysis confirmed that adzuki bean (*V*. *angularis*) is most closely related to rice bean (*V. umbellata*), with an estimated divergence time of 3.1 million years ago (Mya). Comparative genomics revealed large‐scale synteny between the adzuki bean, rice bean, and mung bean genomes, with evidence of interchromosomal rearrangements involving chromosomes 3, 4, and 5 in the adzuki bean lineage. Furthermore, a whole‐genome duplication (WGD) event was detected at ≈56 Mya (Figure S7, Supporting Information), with the 4133 resulting paralogous gene pairs involved in this event enriched in hormone signalling, MAPK signaling, plant‐pathogen interaction, and starch and sucrose metabolism pathways (Figure S8, Supporting Information).

Given the importance of starch as a key nutritional component, we investigated copy number variations in starch metabolism genes across 16 legume species (Table S11, Supporting Information). Most species harbored two copies of *GBSS1* (*LOC107759522*), *SS2* (*LOC107778346*), and *SS3* (*LOC109244492*), while *SS4* (*LOC110098702*) was typically present as a single copy (Figure S9, Supporting Information). Genes encoding starch branching enzymes exhibited broad CNVs, with *SBE2.1* absent in multiple *Vigna* species. Beta‐amylase genes (*BAM*) showed an inverse correlation between copy number and starch content, with the exception of BAM5 and BAM6. Alpha‐amylase and isoamylase genes generally retained single copies. Notably, *BAM5*, which is highly conserved with seven exons, was absent in three *Lupinus* species that exhibit reduced starch accumulation. *BAM6* underwent tandem duplication post‐speciation, resulting in two copies in most *Vigna* species and three copies in *Cajanus cajan* (Figure 1c,d; Table S12, Supporting Information). This dynamic CNV landscape highlights gene duplication as a key evolutionary mechanism shaping starch metabolic pathways in legumes.

### 2.2 Population Structure and Genomic Variation

**Figure 2:**
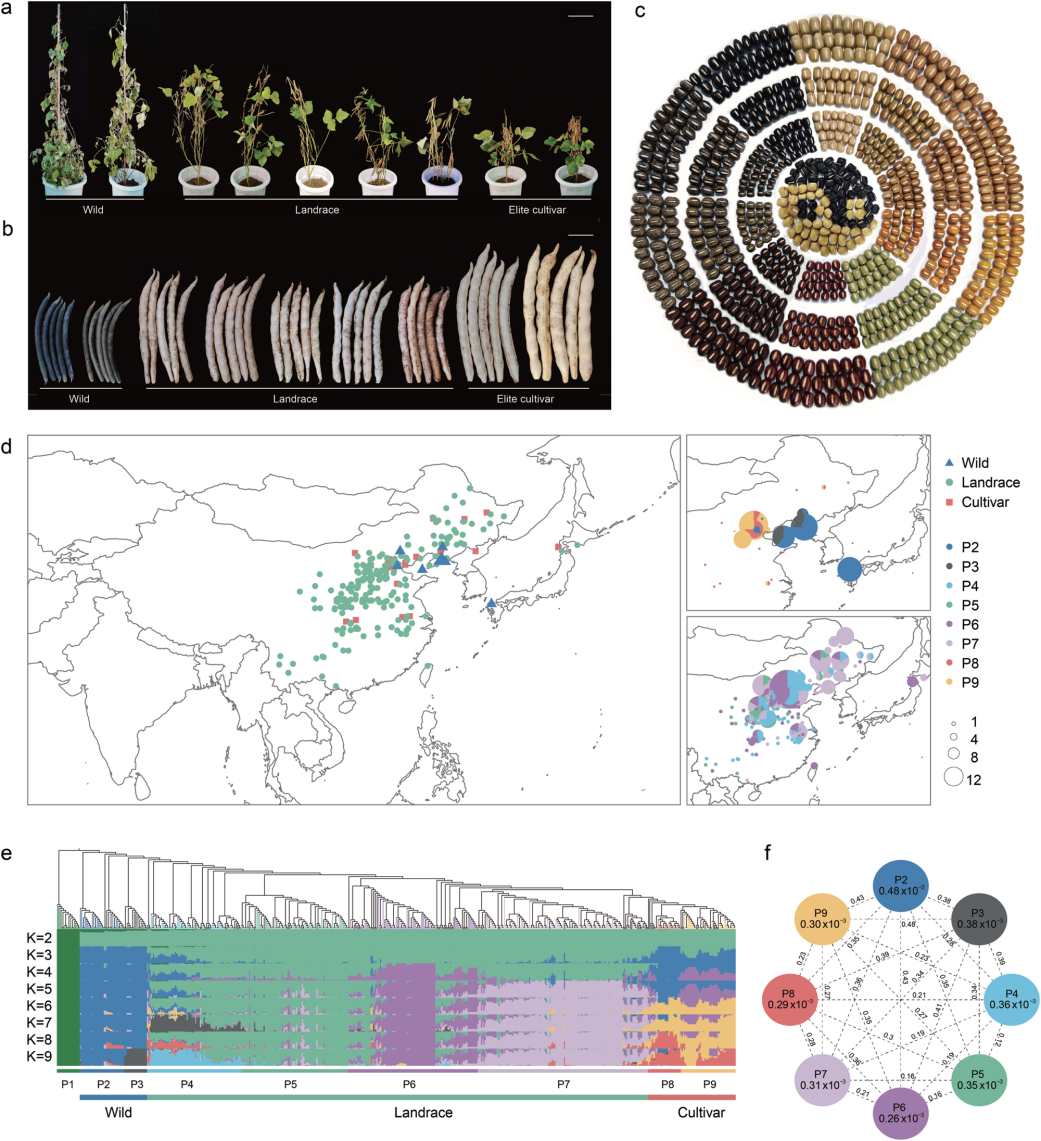
Population structure and genetic diversity of 546 adzuki bean accessions. a–c) Morphological variation in a) plants, b) pods, and c) seeds of wild, landrace, and cultivated accessions. d) Geographic distribution of the 546 accessions, classified into eight populations: P2 (wild), P3 (semi‐wild), P4 (landrace, East/Central China), P5 (landrace, Northwest China), P6 (landrace, North China), P7 (landrace, Northeast China), P8 (elite cultivar, North China), and P9 (elite cultivar, Northeast China). Pie chart size corresponds to the number of samples from each location. e) Phylogenetic tree rooted with 19 wild relatives (left) and ADMIXTURE‐based population structure (*K* = 2–9) for 546 accessions (right). Individuals are colored according to their major ancestry component at *K* = 9. f) Nucleotide diversity (*π*) and population differentiation (*F*_ST_). The *π* value for each population is shown within its circle, and the *F*_ST_ value between populations is shown on the connecting line.

To explore the genetic diversity within adzuki bean populations, we performed whole‐genome resequencing on a core panel of 546 accessions, including 37 wild, 19 semi‐wild, 417 landraces, and 73 modern cultivars (Table S13, Supporting Information). An additional 19 wild relatives were sequenced to serve as an outgroup. The panel showed significant phenotypic differentiation for key agronomic traits, with a clear trend of increased grain size and yield from wild to domesticated forms (**Figure** **2**a–d). Phenotypic data for 13 traits were collected across two growing seasons and three geographical locations. Significant variation among the groups was observed for flavonoid content, hundred‐grain weight, and seed diameter (Figure S10, Supporting Information), indicating that selection during domestication prioritized traits such as higher yield, larger seed size, and modified chemical composition. Using the newly assembled reference genome, we identified 29380944 SNPs and 6170572 InDels. Excluding the wild relatives, the core 546 accessions contained 17974301 SNPs and 3935539 InDels. After filtering SNPs for a minor allele frequency (MAF) ≥ 0.05 and a missing rate ≤ 20%, 3542520 high‐quality SNPs remained for population analysis (Figure S11 and Table S14, Supporting Information).

Population structure analysis, including a neighbor‐joining tree, principal component analysis (PCA), and ADMIXTURE clustering (*K* = 9), revealed eight genetically distinct groups (P2–P9) (Figure 2e). The 19 wild relatives formed a distinct outgroup (P1). Regarding agronomic traits, group P2, comprising the wild accessions, exhibited the lowest yield, poorest seed morphology, fastest linkage disequilibrium (LD) decay (*r* ^2^ = 0.46), highest nucleotide diversity (*π* = 0.48 × 10^−3^), and strong genetic differentiation (*F*_ST_ ranging from 0.28 to 0.43) compared to the other seven groups (Figure 2f). The semi‐wild accessions (P3; *π* = 0.38 × 10^−3^) showed intermediate nucleotide diversity between cultivated and wild adzuki bean, consistent with previous findings.^[^ ^11^ ^]^ The landrace groups (P4–P7) from different regions displayed a trend of increasing yield and improved seed morphology, while the modern cultivated varieties (P8 and P9) had lower nucleotide diversity but superior yield and seed traits (Figures S12,S13 and Tables S15,S16, Supporting Information). These findings indicate that the agronomic traits have progressively improved from wild to cultivated accessions, likely resulting from selective breeding during domestication and improvement processes.

### 2.3 Genomic Signatures of Domestication and Improvement

To identify key genes involved in adzuki bean domestication, we employed a cross‐population composite likelihood ratio (XP‐CLR) analysis. By comparing population pairs (e.g., wild versus landrace and landrace versus cultivar), we identified distinct sets of selective sweeps. This approach revealed 1412 genes under selection during early domestication (wild versus landrace) and 1160 genes under selection during modern improvement (landrace versus cultivar) (Figure S14 and Tables S17–S19, Supporting Information). Furthermore, we identified 538 genes under common selection pressure across both domestication stages, which are likely crucial for long‐term improvements in adzuki bean. Enrichment analysis revealed that these continuously selected genes are closely associated with protein binding and antioxidant activity (Figure S15 and Tables S20–S21, Supporting Information).

**Figure 3:**
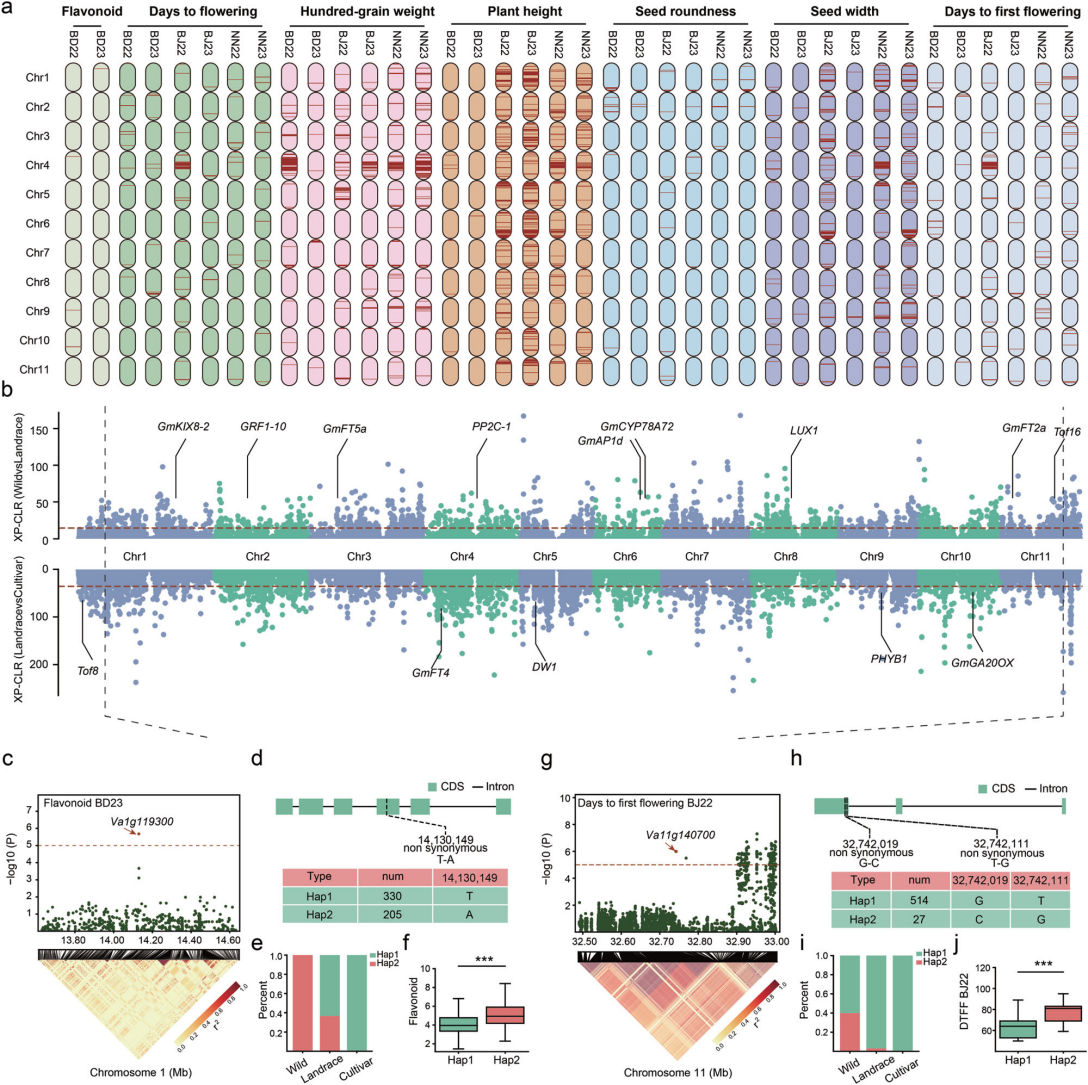
Identification of selection signals and candidate domestication genes. a) Manhattan plots from genome‐wide association studies for seven traits: flavonoid content, days to flowering (DF), hundred‐grain weight (HGW), plant height (PH), seed roundness (SR), seed width (SW), and days to first flowering (DFF). b) Genome‐wide scans for selective sweeps using the XP‐CLR test, comparing landrace versus wild (top) and cultivated versus wild (bottom) populations. c, g) Regional association plots (top) and corresponding LD heatmaps (bottom) for c) flavonoid content and g) days to first flowering. Candidate intervals are marked by orange dashed lines; arrows indicate significantly associated nonsynonymous SNPs. d,h) Gene models and major haplotypes for the candidate genes d) *Va1g119300* and h) *Va11g140700*. Exons are shown as boxes and introns as lines. e, i) Haplotype frequency distributions for e) *Va1g119300* and i) *Va11g140700* across wild, landrace, and cultivated populations. f, j) Phenotypic effects of the major haplotypes of f) *Va1g119300* on flavonoid content and j) *Va11g140700* on days to first flowering. Statistical significance was determined by a Student's *t*‐test (***P < 0.001).

To connect these selection signals to function, we conducted a genome‐wide association study (GWAS)for key agronomic and nutritional quality traits. Based on this analysis, we highlighted eight strong candidate genes associated with seed coat color, flavonoid content, flowering time, grain size, plant height, yield, and seed morphology (Figure S16, Supporting Information). Among the loci associated with seed coat color and flavonoid content (**Figure** **3**a), we identified a region containing the *VaANR1* (*Va1g119300*) gene, which encodes an anthocyanin reductase.^[^ ^11^ ^]^ This locus was also located within the top 5% selection interval in cultivated group 3 (Figure 3b), indicating strong selection pressure during improvement processes. The protein encoded by *Va1g119300* facilitates the conversion of 3‐OH‐anthocyanidins to epi‐flavan‐3‐ols,^[^ ^23^ ^]^ affecting seed coat color. We observed two haplotypes of this gene in the 546 accessions, where accessions carrying Hap2 exhibited higher flavonoid content (Figure 3c,d). The frequency of Hap2 decreased progressively from wild to landrace and then to cultivated populations (Figure 3e,f).

Among the candidate genes associated with flowering, we found a nitrate transporter, *NPF5.4* (*Va11g140700*), which regulates nitrogen levels by participating in nitrate absorption and transport. Plant nitrogen levels are known to be closely linked to flowering time.^[^ ^24^ ^]^ *NRT1.13* (*NPF4.4*) in the *NRT1/PTR/NPF* gene family was also located within the top 5% selection interval in cultivated group 3 (Figure 3b). This gene is required for low‐nitrate acclimation and monitors internal nitrate to regulate shoot architecture and flowering time.^[^ ^25^ ^]^ It harbored two highly significant nonsynonymous variants (Chr11: 32742019, *P*‐value: 1.01 × 10^−6^) and Chr11: 32742111, *P*‐value: 1.01 × 10^−6^), forming two distinct haplotypes associated with flowering time (Figure 3g,h). Hap1 correlated with early flowering and early pod setting, whereas Hap2 was associated with late flowering and late pod setting. The proportion of Hap1 increased progressively from wild to cultivated populations (Figure 3i,j), suggesting that the early flowering and early maturity conferred by Hap1 were selectively favored during adzuki bean domestication.

### 2.4 NAC73 is Associated With Seed Morphology in Adzuki Bean

**Figure 4:**
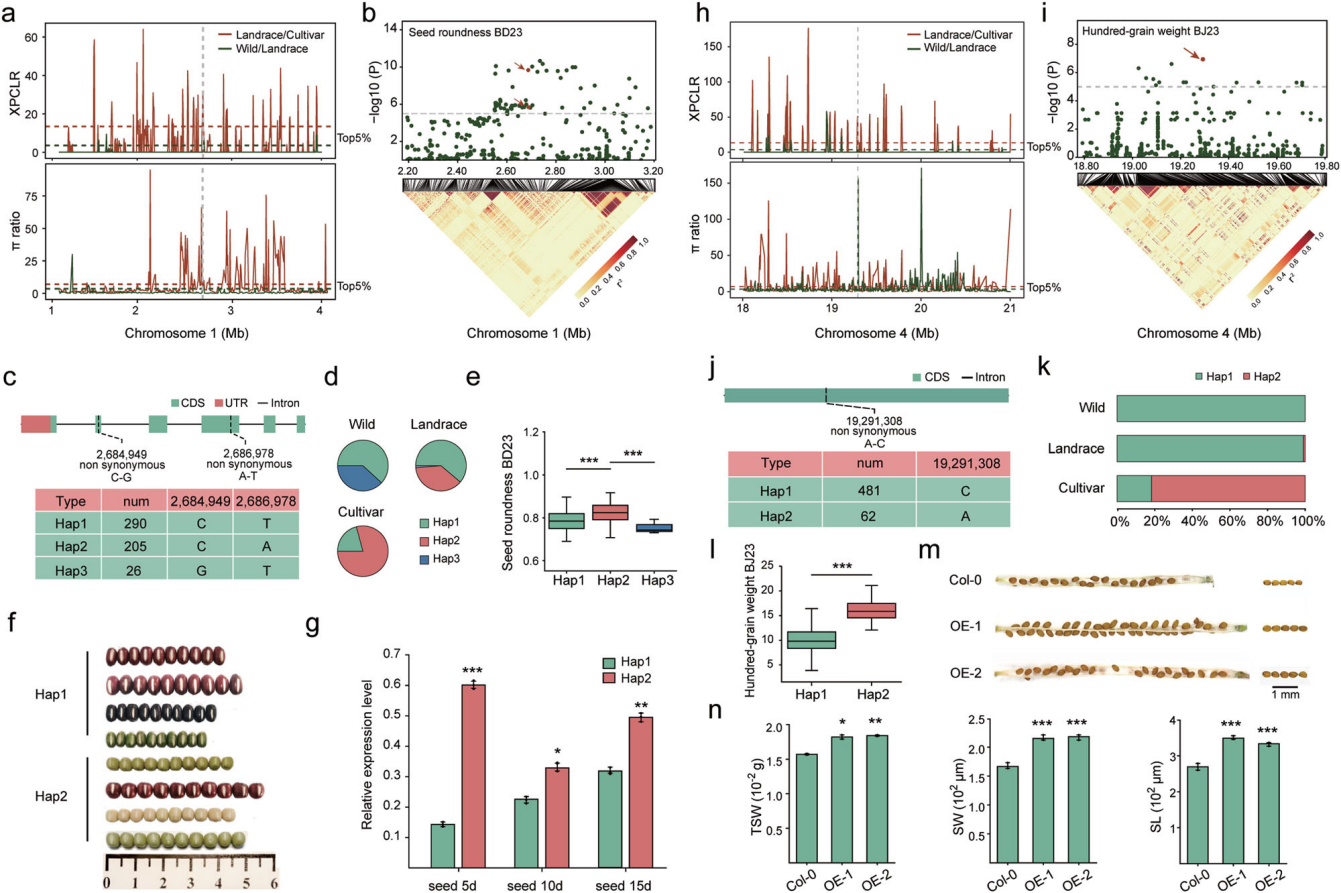
Molecular characterization of candidate genes for seed roundness and hundred‐grain weight. a,h) Selective sweep signals (XP‐CLR and nucleotide diversity ratio) in the genomic regions of a) *Va1g026200* (seed roundness) and h) *Va4g123700* (hundred‐grain weight). b, i) Regional association plots (top) and LD heatmaps (bottom, based on *r* ^2^) for b) seed roundness and i) hundred‐grain weight. c, j) Gene structures and major haplotypes of c) *Va1g026200* and j) *Va4g123700*. d, k) Haplotype frequency distributions of d) *Va1g026200* and k) *Va4g123700* in wild, landrace, and cultivated populations. e) Boxplots comparing seed roundness across major *Va1g026200* haplotypes. f) Representative seed morphology for *Va1g026200* Hap1 and Hap2. Scale bar, 1 mm. g) Relative expression of *Va1g026200* in developing seeds for Hap1 (green) and Hap2 (orange) accessions. m) Silique and seed phenotypes of wild‐type (*Arabidopsis*, Col‐0) and two independent *Va4g123700*‐overexpression lines (OE1, OE2). Scale bar, 1 mm. n) Comparison of thousand‐seed weight (TSW), seed width (SW), and seed length (SL) between wild‐type and OE lines. Data are presented as mean ± SD. Statistical significance was assessed using a Student's *t*‐test (*P < 0.05, **P < 0.01, ***P < 0.001).

Uniform seed size and shape are critical breeding targets for enhancing the commercial value of adzuki bean. As consumer demand for product quality increases, there is a growing preference for varieties with a rounder, more uniform appearance. Using GWAS and selection sweep analysis (Figure S17 and Table S22, Supporting Information), we identified the gene *Va1g026200*, encoding an NAC transcription factor (*NAC73*), that is highly associated with seed roundness. NAC transcription factors are known to affect seed development and are involved in secondary cell wall biosynthesis in *Arabidopsis*.^[^ ^26^ ^]^ This gene showed significant association signals with seed roundness across multiple environments and years and also exhibited strong selection signals between wild and landrace populations, as well as between landrace and cultivated populations (**Figure** **4**a). *NAC73* spans the genomic region from 2.683765 to 2.688120 Mb on Chr1, encompassing two significantly associated non‐synonymous SNP loci (Figure 4b). These two variants delineated three primary haplotypes (Figure 4c). Accessions harboring Hap2 consistently exhibited a significantly higher roundness index compared to those with Hap1 and Hap3. Consistent with positive selection, the prevalence of Hap2 progressively increased across wild, landrace, and cultivated populations, while Hap1 and Hap3 showed a declining trend, with Hap3 nearly absent in landraces (Figure 4d,e; Figure S18, Supporting Information). This pattern indicates a selection bias favoring Hap2 during domestication. Morphologically, accessions with Hap1 exhibited elongated seeds, whereas those with Hap2 displayed a pronounced roundness in seed shape (Figure 4f). Gene expression analysis further revealed that Hap2 exhibited significantly higher expression levels than Hap1 at the 5, 10, and 15 days after pollination (DAP) (Figure 4g). Collectively, the co‐localization of GWAS signals and selective sweeps, combined with haplotype and expression analyses, provides strong evidence that *NAC73* is a key regulator of seed roundness in adzuki bean.

### 2.5 ANKRD50 Regulates Seed Size and Yield Component Traits

Seed size is a primary component of yield and a key target in adzuki bean breeding. Through GWAS and selection analysis for yield improvement, we identified *Va4g123700*, encoding an ankyrin repeat domain‐containing protein (ANKRD50), to be significantly associated with seed size, an important yield component trait. *ANKRD50* exhibited a strong correlation with hundred‐grain weight across multiple environments and also demonstrated evidence of selection between wild and landrace populations, as well as between landrace and cultivated populations (Figure 4h). This gene encodes a protein containing ankyrin repeat domains and harbors one significantly associated non‐synonymous SNP locus (Figure 4i). The two major haplotypes formed by non‐synonymous SNPs were significantly correlated with hundred‐grain weight (Figure 4j). Accessions carrying Hap2 exhibited significantly higher hundred‐grain weight compared to those with Hap1, indicating Hap2 as the favorable haplotype. The increasing frequency of Hap2 in the wild, landrace, and cultivated populations supports its positive selection during domestication (Figure 4k,l; Figure S19, Supporting Information). To validate its function, we overexpressed *ANKRD50* in *Arabidopsis*. The resulting transgenic lines displayed a significant increase in silique length, silique width, seed length, and seed weight (Figure 4m,n; Table S23, Supporting Information). The colocalization of the GWAS peak with selection signals, along with the phenotypic effects of its haplotypes and supporting evidence from heterologous expression, provides strong evidence that *ANKRD50* as a likely causal gene underlying key yield component traits in adzuki bean.

### 2.6 Favorable Alleles and Genomic Selection for Precision Breeding

**Figure 5:**
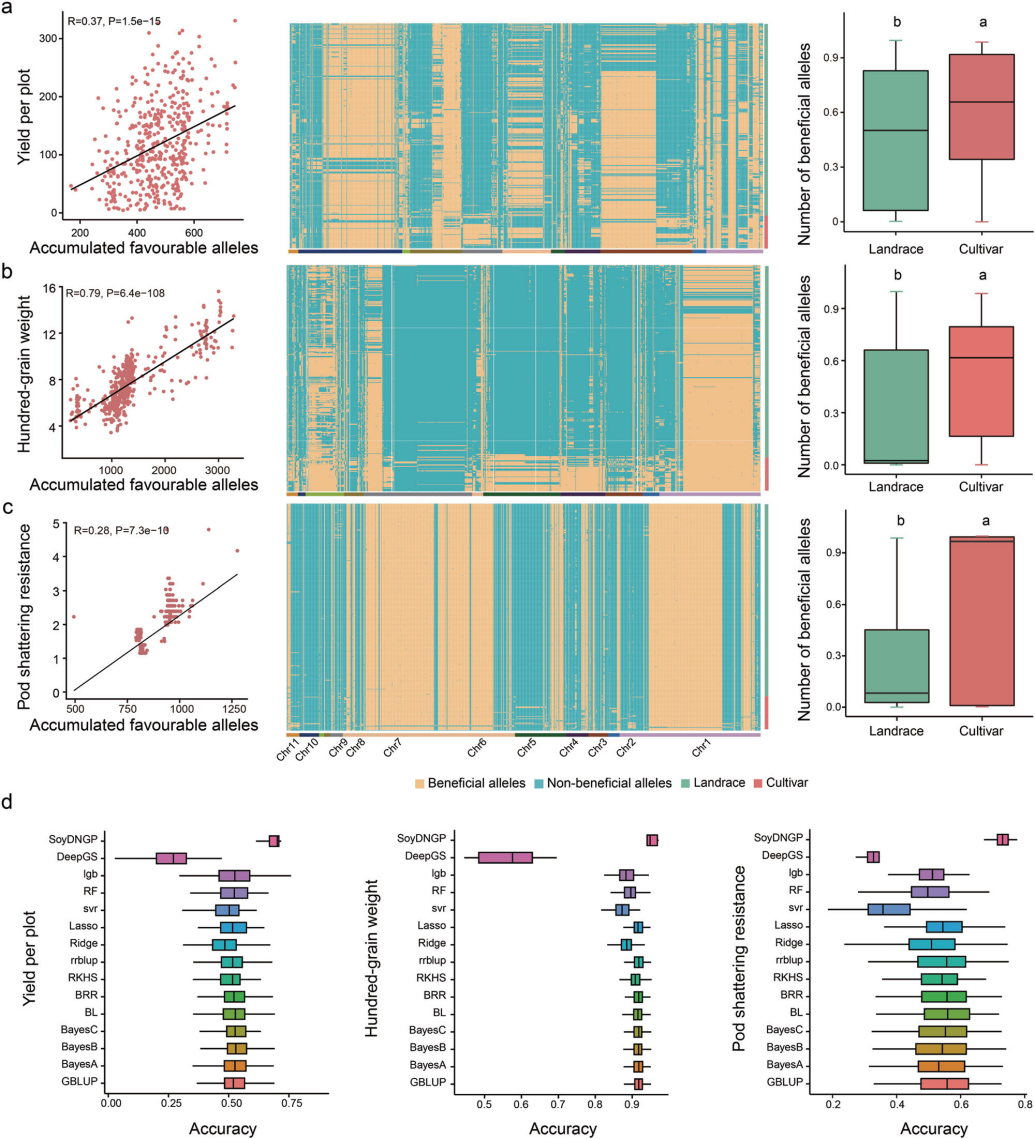
Accumulation of favorable alleles and genomic selection for breeding. a–c) Analysis of favorable alleles for a) Yield Per Plot (YPP), b) Hundred‐Grain Weight, and c) Pod Shattering Resistance (PSR). For each trait: Left panels show the correlation between the number of favorable alleles and phenotypic values (Pearson's *r*). Middle panels display the distribution of favorable alleles across different populations in a heatmap. Right panels compare the frequency of favorable alleles in landrace versus cultivar populations (boxplots). Different letters indicate significant differences by a two‐sided *t*‐test. d) Prediction accuracy (Pearson's *r*) for YPP, HGW, and PSR using 15 different genomic selection models based on favorable alleles. Error bars represent mean ± SD.

Modern adzuki bean breeding has focused on traits, such as increased seed size, early maturity, pod shattering resistance, and high yield. To quantify the genetic basis of this improvement, we identified multiple quantitative trait nucleotides (QTNs) associated with these key agronomic traits from our GWAS results. We then analyzed the allele frequencies of significant SNPs (*P* < 10^−4^) across landrace and cultivated groups. We identified favorable alleles for each QTN and quantified their accumulation. The number of favorable alleles for yield and pod shattering resistance‐related traits generally increased from landrace to cultivated accessions. A progressive increase in the frequency of favorable alleles was observed across the germplasm groups, with the average frequency lowest in landraces and highest in modern cultivars, reflecting strong artificial selection. Furthermore, correlation analysis confirmed a significant positive relationship between the accumulation of favorable alleles and both yield and pod shattering resistance (**Figure** **5**a–c; Figure S20 and Table S24, Supporting Information).

To leverage this genomic information for future breeding, we evaluated the accuracy of GS for these traits. Using 15 different models, prediction accuracies for all traits exceeded 0.5, demonstrating the potential of GS for adzuki bean breeding (Figure 5d). Notably, the SoyDNGP model achieved the highest predictive performance for both hundred‐grain weight and pod shattering resistance, demonstrating the effectiveness of advanced algorithms in capturing complex, non‐additive genetic effects. These findings provide crucial insights for selecting appropriate GS models in future adzuki bean breeding programs.

### 2.7 Accelerating Adzuki Bean Breeding with AdzukiBeanAtlas

**Figure 6:**
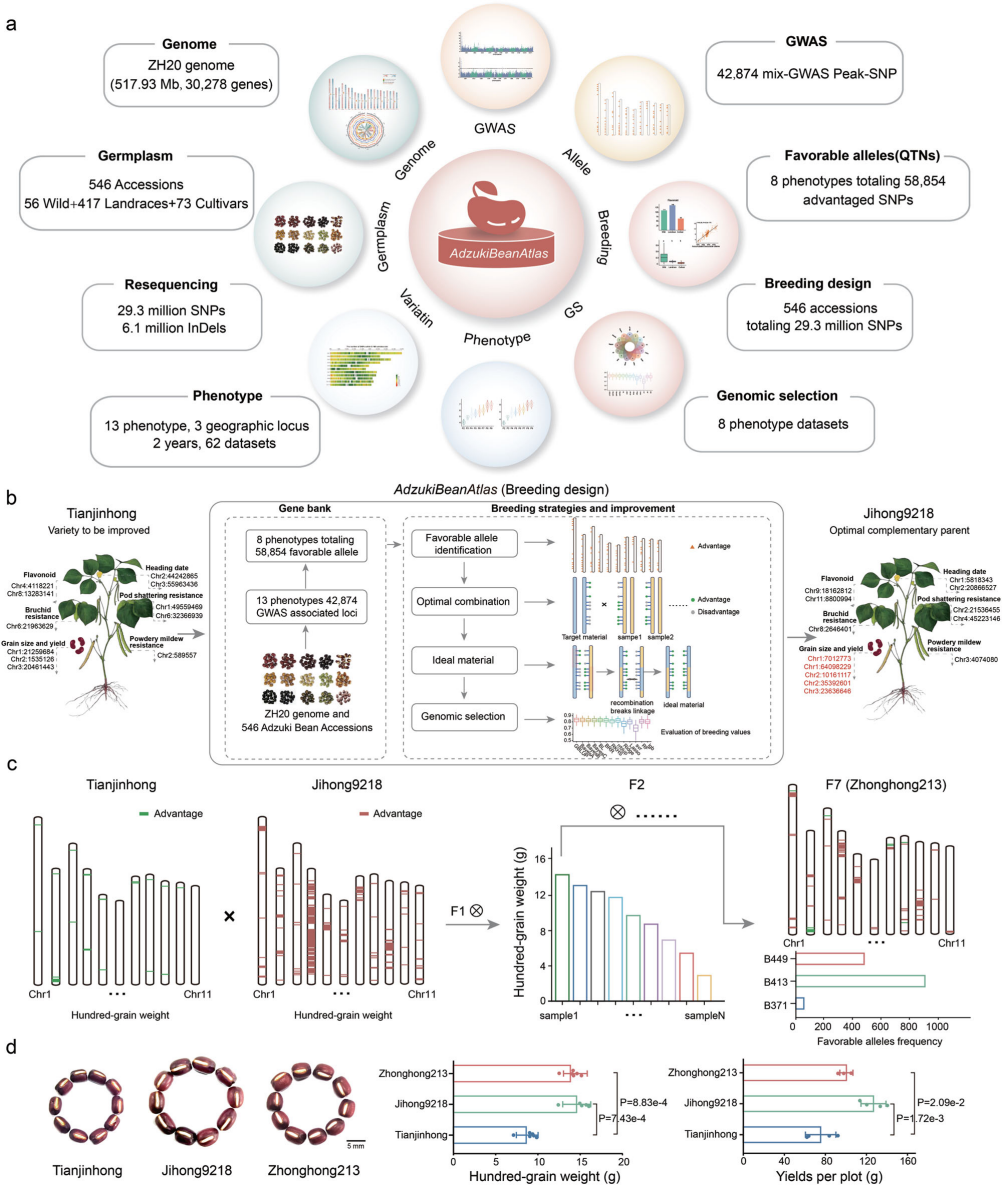
The *AdzukiBeanAtlas* database and its application in molecular breeding. a) Overview of the *AdzukiBeanAtlas* web platform, integrating modules for genomics, germplasm, phenomics, GWAS, variation, and breeding from 546 accessions. b) A schematic workflow of the *AdzukiBeanAtlas* breeding module for targeted trait improvement. c) Marker‐assisted selection strategy using a “Tianjinhong” × “Jihong9218” cross. Introgression and selection of favorable alleles led to the development of the improved F_7_ line “Zhonghong213”. Bar plots illustrate the accumulation of target alleles. d) Phenotypic comparison of hundred‐grain weight (left) and yield per plot (right) among parents (“Tianjinhong”, “Jihong9218”) and the improved line “Zhonghong213”. Data are shown as mean ± SD. Representative grains are shown above the plots. Significance was determined by a two‐tailed Student's *t*‐test. Scale bar, 5 mm.

To translate our extensive genomic and phenotypic data into a practical breeding resource, we developed *AdzukiBeanAtlas*, a comprehensive and publicly accessible platform https://www.cgris.net/AdzukiBeanAtlas. This platform integrates genomic, phenotypic, and genetic analysis data from the 546 adzuki bean accessions (**Figure** **6**a). A central feature of *AdzukiBeanAtlas* is its breeding design module, which facilitates efficient crop improvement by: 1) identifying complementary parents based on favorable allele profiles, 2) simulating optimal allelic combinations at key loci to maximize genetic gain, and 3) predicting the breeding value of simulated crosses using genomic selection.

We demonstrated the practical utility of *AdzukiBeanAtlas* using the adzuki bean landrace “Tianjinhong” as a case study. “Tianjinhong” is valued for its quality traits but is limited by its small seed size. Our goal was to enhance seed size and yield while retaining its elite background. We applied the *AdzukiBeanAtla*s breeding design system to achieve this. First, we identified “Jihong9218” as a complementary parent, as it carries favorable alleles for seed size and yield that are absent in “Tianjinhong”. Next, we simulated optimal combinations of variant loci associated with these target traits. Finally, the potential of the simulated crosses was evaluated using our pre‐trained genomic selection models (Figure 6b).

Following the system's recommendations, we performed a cross between “Tianjinhong” and “Jihong9218”. The resulting progeny were advanced through successive selfing generations, with selection guided by marker‐assisted selection for advantageous loci linked to the target traits (Figure 6c; Table S25, Supporting Information). Phenotypic analysis of the resulting elite line, designated “Zhonghong213”, confirmed significant improvements (Figure 6d; Table S26, Supporting Information). Compared to both parents, “Zhonghong213” exhibited significantly increased seed size and yield while successfully retaining the desirable plant architecture and quality characteristics of “Tianjinhong”. The efficient development of this improved cultivar validates *AdzukiBeanAtlas* as a precise and effective platform for accelerating targeted trait improvement in adzuki bean breeding programs.

## 3 Discussion

Genetic improvement in adzuki bean (*Vigna angularis*), a crop vital for Asian food security, has long been constrained by a narrow genetic base and the lack of foundational genomic resources. Our study directly addresses these critical limitations by providing a high‐quality, chromosome‐scale reference genome for the elite cultivar “ZH20” and a comprehensive genetic variation map from 546 accessions. These resources provide an unprecedented platform for dissecting the crop's genetic architecture and accelerating its improvement.

Our population genomic analyses traced a gradual domestication trajectory from wild relatives to cultivated forms, revealing significant genetic bottlenecks in modern cultivars. Our identification of distinct, geographically structured subpopulations provides a valuable roadmap for breeding and refines our understanding of the crop's domestication history. Recently, a single domestication proposed origin in Japan, suggesting that the high genetic diversity observed in Chinese cultigens is a result of secondary hybridization following an expansion from Japan.^[^ ^27^ ^]^ Our findings, however, support a more complex model. The deep genetic divisions we identified, particularly among landraces from different regions of mainland Asia, are substantial. We propose that these deep genetic divisions are more consistent with a protracted domestication process across a broader geographic range, or potentially multiple independent domestication events, rather than solely the outcome of post‐domestication introgression from a single Japanese source. The discrepancies between these conclusions likely stem from differences in germplasm panels and analytical frameworks. Therefore, our data support the hypothesis that the domestication of adzuki bean may be more reticulated than a single‐origin model suggests, providing a crucial resource for exploring these complex scenarios.

Independent of the precise geographic origins, our data clearly reveal the genetic signatures of selection. A key finding is the progressive enrichment of favorable alleles at identified QTNs from landraces to modern cultivars. This pattern is a classic signature of directional selection, strongly suggesting that these genomic regions have been under persistent artificial selection. This evolutionary validation elevates these QTNs from statistical correlations to high‐confidence targets for breeding.

A central contribution of this work is the identification of robust candidate genes for key agronomic traits. We identified numerous loci associated with key agronomic traits, including heading date, seed coat color, seed shape, seed size, and yield. These findings provide valuable targets for marker‐assisted selection, genomic selection, and future functional genomics research in adzuki bean. Seed size and shape, critical determinants of yield, processing efficiency, and market value,^[^ ^28^, ^29^, ^30^ ^]^ varied considerably across the panel. Integrated GWAS and selection signature analyses identified *ANKRD50* and *NAC73* as strong candidate genes influencing seed size and shape, respectively, which are two primary determinants of yield and market value. Future work should include transgenic complementation by reintroducing the functional *ANKRD50* allele into an adzuki bean mutant or low‐yield accession to further solidify the causal link between this gene and seed size. In *NAC73*, the identified non‐synonymous mutations define haplotypes with distinct seed roundness phenotypes. Notably, the favorable haplotype, Hap2, also exhibits higher expression, suggesting that the phenotypic effect could be driven by changes in both protein function and gene regulation. It remains to be determined whether this effect is primarily due to amino acid substitutions or linked regulatory variations. Further experiments, such as promoter swapping or site‐directed mutagenesis, are needed to decouple these effects. The enrichment of favorable haplotypes for these genes in cultivated germplasm underscores their significance during domestication and improvement.^[^ ^26^, ^31^ ^]^ While homologs of these gene families are known to control organ size and development in other species,^[^ ^28^, ^29^, ^30^ ^]^ their specific functions in adzuki bean are yet to be elucidated. These genes now stand as prime targets for functional validation. While our heterologous expression in *Arabidopsis* provides initial evidence, future studies should employ targeted gene editing techniques, such as CRISPR/Cas9, directly in adzuki bean to definitively confirm their roles in trait regulation and dissect the underlying molecular mechanisms.

Our analysis also provides molecular targets for quality and adaptation traits. Seed coat color is a key quality and domestication trait influenced by flavonoid biosynthesis,^[^ ^32^, ^33^, ^34^, ^35^ ^]^ our analysis implicated *ANR1*, which encodes an anthocyanin reductase, as a candidate gene potentially under strong selection. Future studies should expand haplotype analysis of *ANR1* to include promoter regions and structural variations to capture a wider range of functional diversity. The selection signal at the *ANR1* locus, central to proanthocyanidin biosynthesis, may not only have targeted seed coat appearance but could also be indirectly linked to enhanced abiotic stress tolerance.^[^ ^36^, ^37^ ^]^ This potential dual role of flavonoid pathway genes in both quality traits and stress resilience warrants further investigation, as it could open new avenues for breeding climate‐resilient cultivars. Similarly, the identification of the nitrate transporter gene *NPF5.4* as a candidate for flowering time provides a tangible molecular target for developing varieties better adapted to specific agroecological niches and intensive cropping systems.^[^ ^24^, ^38^ ^]^ These loci represent high‐value targets for marker‐assisted selection and future functional validation.

A major challenge in modern breeding is the effective translation of vast genomic datasets into practical, actionable strategies. Our development of the *AdzukiBeanAtlas* platform directly addresses this gap. As demonstrated by the successful enhancement of the “Tianjinhong” landrace, our platform moves beyond traditional trial‐and‐error methods towards a more precise, “design‐led” breeding paradigm. This approach not only accelerates the breeding cycle but also significantly increases the probability of achieving desired trait combinations, providing a powerful new tool for the adzuki bean community.

A key innovation of *AdzukiBeanAtlas* is its seamless integration of GWAS, QTN mapping, and GS into a single, user‐friendly workflow. This systems‐based approach facilitates the holistic improvement of multiple traits simultaneously, as demonstrated by our ability to increase yield and seed size in “Zhonghong213” while preserving the elite quality traits of “Tianjinhong”. Looking forward, a key frontier is to move beyond predictive accuracy toward biological interpretability. Future work should focus on integrating our GS models with pathway analyses and gene regulatory networks. This would help decipher the biological mechanisms underlying genomic predictions, transforming GS from a powerful statistical tool into a mechanistically informative approach that deepens our fundamental understanding of complex traits.

In conclusion, the genomic resources, trait associations, candidate genes, and the *AdzukiBeanAtlas* platform presentedin this work collectively establish a robust foundation for accelerating the development of improved adzuki bean varieties. While functional validation of candidate genes and further exploration of genotype‐by‐environment interactions remain important future steps, this work provides the critical tools needed to guide the precision breeding of climate‐resilient adzuki bean cultivars designed to meet future agricultural demands and consumer preferences.

## 4 Experimental Section

### Plant Materials and Germplasm Panels

The adzuki bean accessions used in this study were obtained from the National Crop Genebank of China, Chinese Academy of Agricultural Sciences (CAAS). For genome sequencing and *de novo* assembly, young leaves from a single plant of the elite Chinese cultivar “ZH20” were collected. For population‐level analyses, a total of 546 adzuki bean accessions, comprising 56 wild accessions, 417 landraces, and 73 modern cultivars, were sequenced. To provide a robust outgroup for phylogenetic analysis, an additional 19 wild accessions were sequenced, bringing the total number of sequenced samples to 565. Unless otherwise specified, all population genetic, GWAS, and downstream analyses were conducted on the core panel of 546 accessions. Detailed information, including the geographic origin of these samples, is provided in Table S13 (Supporting Information).

### DNA/RNA Isolation and Sequencing

Tender leaves were sampled for DNA extraction and immediately frozen in liquid nitrogen. A total of 1.5 µg of DNA per sample was used to generate sequencing libraries using the Truseq Nano DNA HT Sample Preparation Kit (Illumina, CA, USA) according to the manufacturer's recommendations. The multiplexing barcode was added to attribute the sequences of each sample. Libraries were prepared by randomly interrupting genomic DNA to a fragment size of ≈350 bp using a Covaris crusher, followed by end‐polishing, addition of a polyA tail, and ligation with full‐length adapters for Illumina sequencing, followed by further PCR amplification. PCR products were purified using the AMPure XP Bead System. The library size distribution was analyzed using the Agilent2100 bioanalyzer and quantified using real‐time PCR. The Illumina HiSeq X sequencing platform was used to generate ≈7.1 Tb of raw sequences. For sequencing the genome of “ZH20”, a 15 Kb library was constructed and sequenced using the PacBio Sequel II platform (Pacific Biosciences, CA, USA), generating a total of 41.36 Gb CCS HiFi reads with an N50 size of 22.31 Kb using CCS software (v3.0.0) (https://github.com/pacificbiosciences/unanimity/) using parameters “–min‐passes 3 –min‐length 10000 –max‐length 1000000 –min‐rq 0.99”. Additionally, Hi‐C libraries were constructed using fresh leaf tissue fixed in 1% formaldehyde to extract chromatin, which was digested using the DpnII restriction enzyme (New England Biolabs, USA). After quality control, the Hi‐C libraries were sequenced on an Illumina NovaSeq X platform (Illumina, CA, USA), yielding a total of 72.17 Gb Hi‐C data. For transcriptome sequencing to aid in gene prediction, five samples from different tissues, including root, stem, leaf, flower, and fresh seeds, were collected and stored at −80 °C. Total RNA was isolated using the RNAprep Pure Plant Kit (TIANGEN, Beijing, China) to construct RNA‐seq libraries and sequenced on the Illumina NovaSeq Xten platform (Illumina, CA, USA). A total of 63.89 Gb paired‐end reads were generated for the seven RNA‐seq libraries (Table S1, Supporting Information).

### Genome Assembly

To estimate the genome size of adzuki bean, a *k*‐mer analysis was performed with Jellyfish (v2.2.7) using the Illumina short reads.^[^ ^39^ ^]^ The contigs of “ZH20” were assembled using PacBio HiFi reads using hifiasm,^[^ ^40^ ^]^ with parameters setting “‐l 0 ‐k 51 ‐w 51 –write‐paf –write‐ec”. Contigs were anchored into chromosomes using Hi‐C data. Hi‐C reads were aligned to HiFi contigs using HICUP (v0.7.3),^[^ ^41^ ^]^ yielding an alignment BAM file. Then, contigs were clustered into scaffolds using ALLHiC.^[^ ^42^ ^]^ The assembled scaffolds were manually corrected with Juicebox (v1.11.08).^[^ ^43^ ^]^ Benchmarking Universal Single‐Copy Orthologs (BUSCO)^[^ ^44^ ^]^ was used to determine assembly completeness based on the Embryophyta (odb10) database.

### Genome Annotation

*De novo* and homology‐based techniques were used for the identification of repetitive elements. Similarity searches were performed against the RepBase database (http://www.girinst.org/repbase/) using RepeatProteinMask. Next, LTR_FINDER,^[^ ^45^ ^]^ RepeatScout, and RepeatModeler (http://www.repeatmasker.org/RepeatModeler.html/) were used to build a *de novo* library for *de novo* annotation. The *de novo* library along with repeats identified from homology‐based evidence was used to screen the genome for repeats using RepeatMasker (http://repeatmasker.org/). A comprehensive strategy combining ab initio prediction, protein‐based homology searches, and RNA sequencing was used to predict gene structure. Protein sequences from closely related species including, *V. angularis*, *V. radiate*, *V. unguiculata*, *V. umbellata*, *P. vulgaris*, and *G. max* were aligned to the corresponding genome using WUblast^[^ ^46^ ^]^ with an E‐value cutoff of 10^−5^, and hits were combined by Solar software.^[^ ^47^ ^]^ GeneWise was used to predict the exact gene structure of the corresponding genomic regions for each WUblast hit.^[^ ^48^ ^]^ The gene structure predicted by GeneWise was denoted as Homo‐set (homology‐based prediction gene set). Five ab initio gene prediction programs Augustus (version 2.5.5),^[^ ^49^ ^]^ Genscan (version 1.0),^[50^^]^ Geneid,^[^ ^51^ ^]^ GlimmerHMM (version 3.0.1)^[^ ^52^ ^]^ and SNAP^[^ ^53^ ^]^ were used to predict coding regions in the repeat‐masked genome. RNA‐seq data were mapped to the assembly using Tophat (version 2.0.8)^[^ ^54^ ^]^ and Cufflinks (version 2.1.1),^[^ ^55^ ^]^ then used to assemble transcripts into gene models (Cufflinks‐set). Additionally, gene models were predicted from Trinity‐assembled transcripts by PASA,^[^ ^56^ ^]^ and denoted as PASA‐T‐set (PASA Trinity set). Gene model evidence from the Homo‐set, Cufflinks‐set, PASA‐T‐set, and ab initio programs was combined by EVidenceModeler (EVM)^[^ ^57^ ^]^ into a non‐redundant set of gene annotations. Weights for each type of evidence were set as follows: PASA‐T‐set > Homo‐set > Cufflinks‐set > Augustus > GeneID = SNAP = GlimmerHMM = Genscan. The predicted protein sequences were assigned putative functions by performing similarity searches using BLAST against five protein/function databases: NCBI‐NR, InterPro, Gene Ontology, the Kyoto Encyclopedia of Genes and Genomes (KEGG)^[^ ^58^ ^]^ and Swiss‐Prot^[^ ^59^ ^]^ using E‐value cutoff of 1 × 10^−5^. The Gene Ontology terms were assigned using InterProScan^[^ ^60^ ^]^ with parameters: “‐f TSV ‐dp ‐gotermes ‐iprlookup”. To identify telomeric regions in the genome, telomere detection was performed using the tidk software (version 0.2.0).^[^ ^61^ ^]^ The known telomeric repeat sequence (“AAACCCT”) was searched for. The identified regions are detailed in Tables S4 and S5 (Supporting Information).

### Gene Family Clustering Analysis

For gene family analysis, protein sequences from a total of 16 species, including *A*. *thaliana*, *V*. *unguiculata*. *unguiculata*, *V*. *unguiculata*. *sesquipedalis*, *V*. *radiata*, *V*. *angularis*, *V*. *umbellata*, *V*. *subterranea*, *P*. *vulgaris*, *G*.*max*, *C*. *cajan*, *P*. *sativum*, *M*. *truncatula*, *C*. *arietinum*, *L*. *japonicus*, *L*. *angustifolius*, and *L*. *albus* were used to identify orthologs using Orthofinder.^[^ ^62^ ^]^

### Phylogenetic Tree Construction and Divergence Time Estimation

A total of 541 single‐copy orthologs from the 16 species were used to construct a phylogenetic tree. The protein sequences from each species were concatenated into 16 muscle sequences using the MUSCLE software.^[^ ^63^ ^]^ A maximum‐likelihood tree was constructed with RAxML (v8.0.19).^[^ ^64^ ^]^ with 100 bootstrap replicates. Divergence times were estimated under a relaxed clock model using the Mcmctree program in PAML (v4.9e).^[^ ^65^ ^]^ The “JC69” model in Mcmctree was run with a sample number set to 1000000 and burn‐in iteration set to 10000. Calibration points were obtained from the TimeTree database (http://www.timetree.org/).

### Expansion and Contraction of Gene Family and Whole Genome Duplication Analysis

Gene family evolution was identified as a stochastic birth and death process using CAFE (version 4.2) software.^[^ ^66^ ^]^ The phylogenetic tree topology and branch lengths were considered to infer the significance of the change in gene family size for each branch. The ortholog gene pairs were identified using MCScanX.^[^ ^67^ ^]^ Calculate_4DTV_correction.pl was used to calculate 4dtv between gene pairs, and synonymous_calc.py (https://github.com/tanghaibao/bio‐pipeline/tree/master/synonymous_calculation/) was used to calculate *Ks*. Both 4dtv and *Ks* results indicated that two WGD events occurred very closely.

### Identification of Beta‐Amylase Genes

The beta‐amylase gene in *A. thaliana* (https://www.arabidopsis.org/browse/gene_family/GlycosideHydrolase/) was used to perform a similarity search using BLAST against the 16 species to identify the copy number of beta‐amylase genes in each species. Gene groups of different classes were aligned using muscle, and an evolutionary tree was constructed using treebestnj.^[^ ^68^ ^]^ Starch and amylose contents data were from the Nutrient Data Laboratory, ARS, USDA National Food and Nutrient Analysis Program Wave 6m, 2002 Beltsville MD.^[^ ^69^ ^]^

### Whole Genome Resequencing Quality Control, Variation Calling, and Annotation

The raw sequencing data from whole genome resequencing were subjected to quality control to remove the low‐quality reads including: i) reads with ≥10% unidentified nucleotides (N); ii) reads with >10 nt aligned to the adaptor, with ≤10% mismatches allowed; iii) reads with >50% bases having phred quality <5; and iv) putative PCR duplicates. Consequently, ≈7.1 Tb (≈13 Gb per sample) high‐quality genomic data were used. FASTP^[^ ^70^ ^]^ was used for quality control to obtain clean reads for subsequent analysis (Table S14, Supporting Information). Clean reads for each sample were aligned to the assembled “ZH20” genome using BWA (v0.7.8) with default parameters (“mem‐k32‐M‐R”).^[^ ^71^ ^]^ To reduce mismatches caused by PCR amplification before sequencing, Samtools (v0.1.19) was used to sort and deduplicate the alignments was used.^[^ ^72^ ^]^ The Genome Analysis Toolkit (GATK v4.2) was used to identify variations, including SNPs and InDels.^[^ ^73^ ^]^ The raw variants were filtered to retain SNPs with variant quality >30, MAF ≥0.05, and missing rate ≤20%. A total of 3542520 high‐quality SNPs were retained. SNPs and indels were further annotated using ANNOVAR (v2013‐05‐09).^[^ ^74^ ^]^

### Population Structure Analysis

To obtain a set of markers suitable for population structure analysis while minimizing bias from linkage disequilibrium, the full SNP set was pruned using PLINK (v1.90)^[^ ^75^ ^]^ with the parameters ′–indep‐pairwise 50 1 0.2′. This standard procedure removes SNPs in high LD, resulting in a set of 302372 relatively independent SNPs that were used for phylogenetic tree and ADMIXTURE analysis. The individual‐based neighbor‐joining tree was constructed based on the *p*‐distance using TreeBest (v1.9.2)^[^ ^68^ ^]^ with 1000 bootstrap replications. Population genetic structure was examined using ADMIXTURE (v1.23),^[^ ^76^ ^]^ specifying *K* ranged from 2 to 15. Principal component analysis was conducted using GCTA (v1.93.2).^[^ ^77^ ^]^ VCFtools (v1.15)^[^ ^78^ ^]^ was used to calculate nucleotide diversity and inter‐population fixation statistics (*F*_ST_) values for each subpopulation, with a window size of 10 Kb and step size of 5 Kb. Linkage imbalance analysis was calculated for all SNPs up to 500 Kb using the PopLDdecay (v3.40)^[^ ^79^ ^]^ parameter (‐MaxDist 500 ‐MAF 0.05 ‐Miss 0.2). The BLUE value of the phenotype was calculated using the R package lme4,^[^ ^80^ ^]^ and a box plot was generated with the ggplot2 package in R.^[^ ^81^ ^]^

### Selection Sweep Analysis

The cross‐population composite likelihood ratio was calculated using XP‐CLR software (v1.0)^[^ ^82^ ^]^ employing a sliding window of 10 Kb and a step size of 5 Kb. Selective sweeps were identified by comparing differential scans in the wild group with landrace groups, as well as between landrace and cultivated groups. The nucleotide diversity (*π*) was calculated using VCFtools in 20 Kb sliding windows with a 10 Kb step. The fixation statistics (*F*_ST_) between different populations were calculated using a set of Python scripts (https://github.com/simonhmartin/genomics_general/popgeneWindows.py/) with the parameters set as “‐w 100000, ‐s 10000, ‐f haplo”. Genes in the top 5% of these regions were considered candidates for selection.

### KEGG and GO Enrichment Analysis

The GO and pathway annotation was performed using the R packages KEGGREST^[^ ^83^ ^]^ and GoDB^[^ ^84^ ^]^ based on the protein sequences of the adzuki bean genome. GO and KEGG enrichment analysis was conducted using agriGO (v2.0), and Kofam KOALA (v1.3.0), respectively.^[^ ^85^ ^]^ The significance of enrichment was assessed using the Fisher test. Results with more than five annotations and a Bonferroni‐corrected false discovery rate of < 0.05 were marked as significant and visualized using the R package ClusterProfiler (v3.10.0).^[^ ^86^ ^]^

### Phenotyping for Agronomic Traits

The 546‐accession panel was phenotyped for 13 agronomic traits across three different locations: Beijing (40.23°N, 116.56°E), Nanyang (32.98°N, 112.52°E), and Nanning (23.15°N, 108.28°E) over three years. For each planting, 60 seeds per landrace were sown in two rows (30 plants per row). Phenotypic data were collected for traits related to flowering time, plant architecture, and pod characteristics, and seed size and yield. Pod length, width, and height were measured using a vernier caliper on at least five healthy plants from each landrace post‐harvest. Seed traits, including total seed number per plant, total number of pods per plant, pod width, pod length, and seed coat color, were also evaluated. Seed length, width, diameter, hundred‐grain weight, total seed surface area, seed area expansion ratio, average seed area, seed area standard deviation, and average seed circumference were measured using an automatic seed counting and analyzing instrument (Model SC‐G, Wanshen Co. Ltd., China). Yield per plot were measured by harvesting, threshing, and air‐drying each plot separately until the moisture content of the seeds reached 12%–14%, followed by weighing. Phenotypes in all three environments were investigated following the “Descriptors and data standards for adzuki bean [*Vigna angularis* (Willd) Ohwi & Ohashi]”.^[^ ^87^ ^]^

### GWAS Analysis

GWAS was conducted using 3542520 high‐quality SNPs and the mixed linear model in GEMMA (v0.98.3).^[^ ^88^ ^]^ The genetic relationship (*K*) matrix was calculated using GEMMA, while PCA was calculated with GCTA to serve as a covariate for GWAS correction. The significance threshold was determined by a Bonferroni correction for the effective number of independent markers^[^ ^89^ ^]^ (*n* = 302372), resulting in a genome‐wide significance level of *P* < 3.31 × 10^−6^. The local LD blocks with 63 Kb (*r* ^2^ = 0.38) around peak SNPs (above the threshold) were defined as the candidate associated regions. Genes within the candidate associated regions were selected as the candidate genes for the GWAS associations, based on selective sweeps, variant annotation, and aided by homologous and functional annotations from *Arabidopsis thaliana* and other model crops (Tables S22, Supporting Information). Haploview (v4.2) was used to detect local linkage disequilibrium,^[^ ^90^ ^]^ and haplotype analysis was conducted to explore the association between haplotypes and phenotypes.

### RNA Isolation and RNA Sequencing

RNA sequencing was performed on the developing seeds of “ZH20” (cultivar) and B511 (wild) accessions at the 5, 10, and 15  DAP, with three biological replicates. Total RNA was extracted using the RNeasy Plant Mini Kit (Qiagen, China), and sequencing was conducted on the Illumina X platform, generating ≈6 Gb of 150‐bp paired‐end reads per sample. Clean reads were aligned to the reference genome using HISAT2 (v2.2.1).^[^ ^91^ ^]^ Gene expression levels were quantified using featureCounts (v1.5.0,^[^ ^92^ ^]^ and analyzed with edgeR (v3.14.0).^[^ ^93^ ^]^

### RNA Extraction and Quantitative PCR Analysis

Quantitative real‐time PCR (qRT‐PCR) was employed to quantify *NAC73* expression levels in seeds at the same DAP stages. Total RNA was extracted using Trelief RNAprep Pure Plant Kits (Polysaccharides & Polyphenolics‐rich, Tsingke, China), and cDNA synthesis was performed using the PrimeScript RT Reagent Kit with gDNA Eraser (Takara, Japan). qRT‐PCR was conducted using TSINGKE Master qPCR Mix (SYBR Green I with UDG) on a StepOnePlus Real‐Time PCR System (Applied Biosystems, USA), following the manufacturer's protocol. *ACTIN* was used as a reference gene, and data were analyzed using the 2^−ΔΔCT^ method. Each qRT‐PCR assay included three technical replicates per biological sample, with a minimum of three biological replicates. Primers were designed to span an intron to prevent genomic DNA amplification (Table S23, Supporting Information).

### Arabidopsis Transformation

To investigate the function of the candidate yield gene *ANKRD50*, its coding sequence (favorable haplotype, Hap2) was cloned from “ZH20” cDNA. The full coding sequence of *ANKRD50* was amplified and cloned into the pEasy‐T1 vector, which was subsequently subcloned into the binary vector pCambia1302 under the control of the Cauliflower mosaic virus (CaMV) 35S promoter. The *35S*::*ANKRD50*‐Hap2 construct was transformed into *Agrobacterium tumefaciens* (strain GV3101) and subsequently into *Arabidopsis thaliana* ecotype Columbia (Col‐0) via the floral dip method. *ANKRD50* expression was quantified in primary inflorescence stems of two‐week‐old T1 transgenic plants using qPCR. The two lines showing the highest *ANKRD50* expression were selected for further analysis. Phenotypic analysis was conducted on T3 homozygous plants. Primers used in these experiments are listed in Table S23 (Supporting Information).

### Genomic Selection and Breeding Platform

Breeding‐favorable genotypes were defined based on previous studies,^[^ ^94^ ^]^ utilizing the favorable SNP effects in significant association signals. Genotypic effects were assessed by calculating the average phenotypic values of homozygous genotypes. For yield‐related traits, genotypes correlated with higher yields were considered favorable. SNPs associated with yield, maturity, resistance, and pod dehiscence were analyzed for breeding‐favorable genotypes. The ggplot2 R package was used to visualize subpopulation accumulation and favorable genotypes, and ComplexHeatmap was used to plot their distribution across the genome.^[^ ^95^ ^]^ To evaluate the impact of favorable genotypes on phenotypic prediction, multiple genome prediction models, including BayesA, BayesB, BayesC,^[^ ^96^ ^]^ BL, BRR, RKHS, GBLUP, rrBLUP, Ridge, Lasso,^[^ ^97^ ^]^ SVR, RF,^[^ ^98^ ^]^ DeepGS and SoyDNGP were tested using the BGLR (v1.0.8).^[^ ^99^ ^]^ Cross‐validation was performed by randomly selecting 20% of the material as the testing population, training the model, and predicting phenotypic values in the test population across 100 replicates. These resources were integrated into *AdzukiBeanAtlas* (https://www.cgris.net/AdzukiBeanAtlas/), a web platform featuring a breeding design module. The module's workflow is as follows: 1) Parental Analysis: A user uploads genomic data for a recipient parent, and the system identifies unfavorable alleles at known QTNs. 2) Trait Selection: The user selects target traits for improvement. 3) Donor Recommendation: The system screens the 546‐accession database to recommend donor parents with complementary favorable alleles. 4) Progeny Simulation and Prediction: The module simulates the genotypes of potential progeny and predicts their genomic estimated breeding values (GEBVs) using the pre‐trained GS models.

## Conflict of Interest

The authors declare no conflict of interest.

## Author Contributions

L.H., X.W., Y.Z., and V.G. contributed equally to this work. H.C. designed and completed the project. Y.C., L.W., S.W., and X.C. provided materials and information. C.L., Y.W., W.G., G.L., X.Z., and X.C. managed the fieldwork and prepared the samples. L.H., X.W., X.Y., R.X., H.Z., Y.C., and W.G. contributed to phenotyping. R.C., T.C., and Q.S. contributed to conducting experiments. H.C., Y.Z., D.G., X.J., R.B., and C.J. performed bioinformatics and data analysis. H.C., and Y.Z. wrote the paper with inputs from all authors, and H.C., V.G., C.J., and R.K.V. revised and finalized the manuscript. All authors read and approved the final manuscript.

## Supporting information



Supporting Information

Supplemental Table

## Data Availability

The data that support the findings of this study are openly available in Genome Sequence Archive (GSA) database at https://ngdc.cncb.ac.cn, reference number 91.
