# Correction to “High‐Resolution Genomic Resources for Trait Mapping and Precision Breeding for Adzuki Bean (*Vigna angularis*)”

**DOI:** 10.1002/advs.77645

**Published:** 2026-10-08

L. Hu, X. Wang, Y. Zhang, et al. “High‐Resolution Genomic Resources for Trait Mapping and Precision Breeding for Adzuki Bean (*Vigna angularis*).” *Advanced Science* 13, no. 4 (2026): e07157, https://doi.org/10.1002/advs.202507157.

In the “Data Availability Statement” section, the previous information regarding the Genome Sequence Archive (GSA) was incorrect. The correct accession numbers for the ZH20 genome assembly and raw sequencing records is PRJCA028848, which is available through the National Genomics Data Center (NGDC) at https://ngdc.cncb.ac.cn.

We apologize for this error.

