# Supplementary material for: High‐Resolution Genomic Resources for Trait Mapping and Precision Breeding for Adzuki Bean (Vigna angularis)
Source: Adv Sci (Weinh). 2025 Nov 19;13(4):e07157. doi: 10.1002/advs.202507157 (PMC12822460; doi:10.1002/advs.202507157)
Supplement: Supplementary file 1 — Supporting Information [file ADVS-13-e07157-s002.pdf]

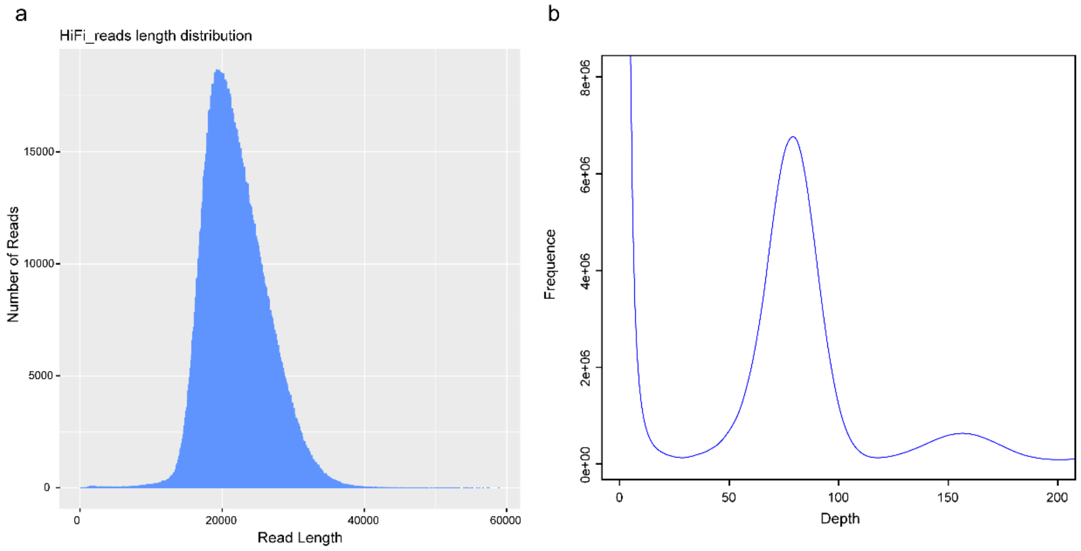

Supplementary Figure 1. Genome size estimation and sequence quality assessment for 'ZH20'.

a) Distribution of PacBio HiFi read lengths and quality scores, demonstrating the high quality of the raw sequencing data. **b)** K-mer frequency distribution (k=21) used to estimate the 'ZH20' genome size. The analysis resulted in a predicted genome size of approximately 517.93 Mb.

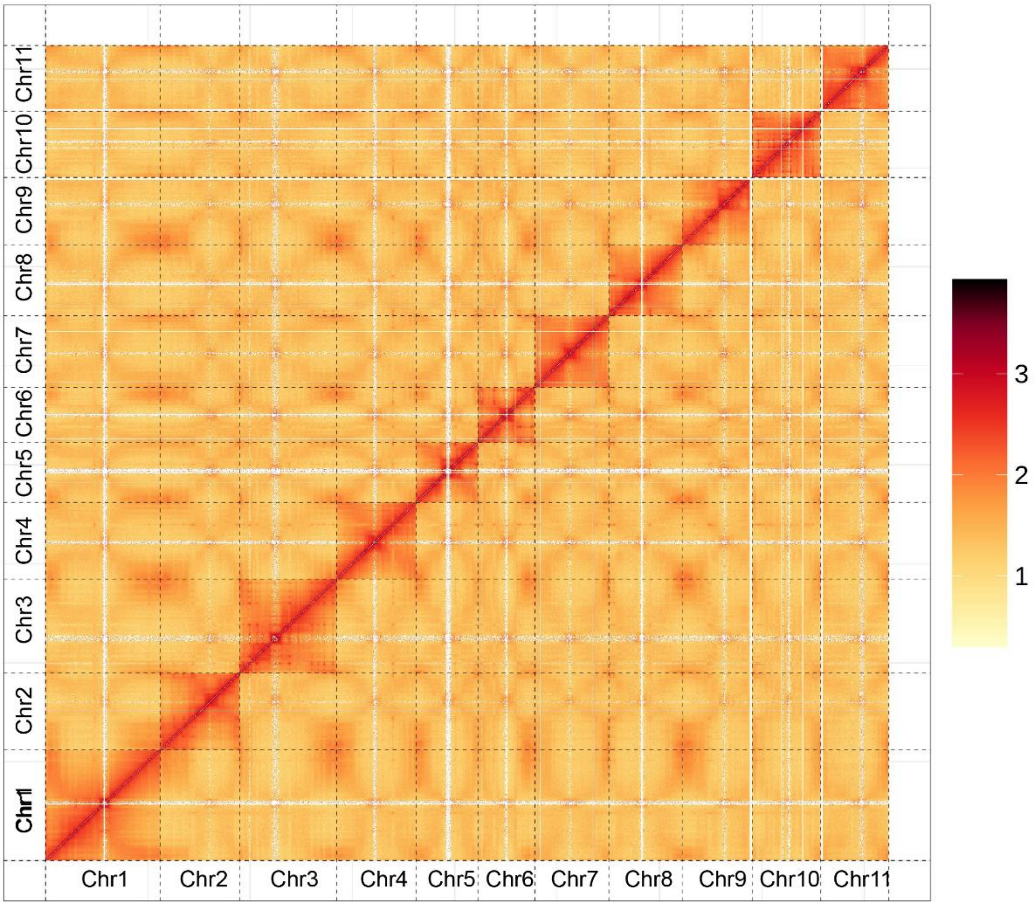

Supplementary Figure 2. Hi-C interaction heatmap for the 'ZH20' genome assembly. The heatmap visualizes the frequency of chromosomal interactions across the genome. The strong diagonal signal for each of the 11 pseudochromosomes indicates a high-quality, chromosome-level assembly with correct scaffold ordering and orientation. Interaction density was calculated in 51 kb windows.

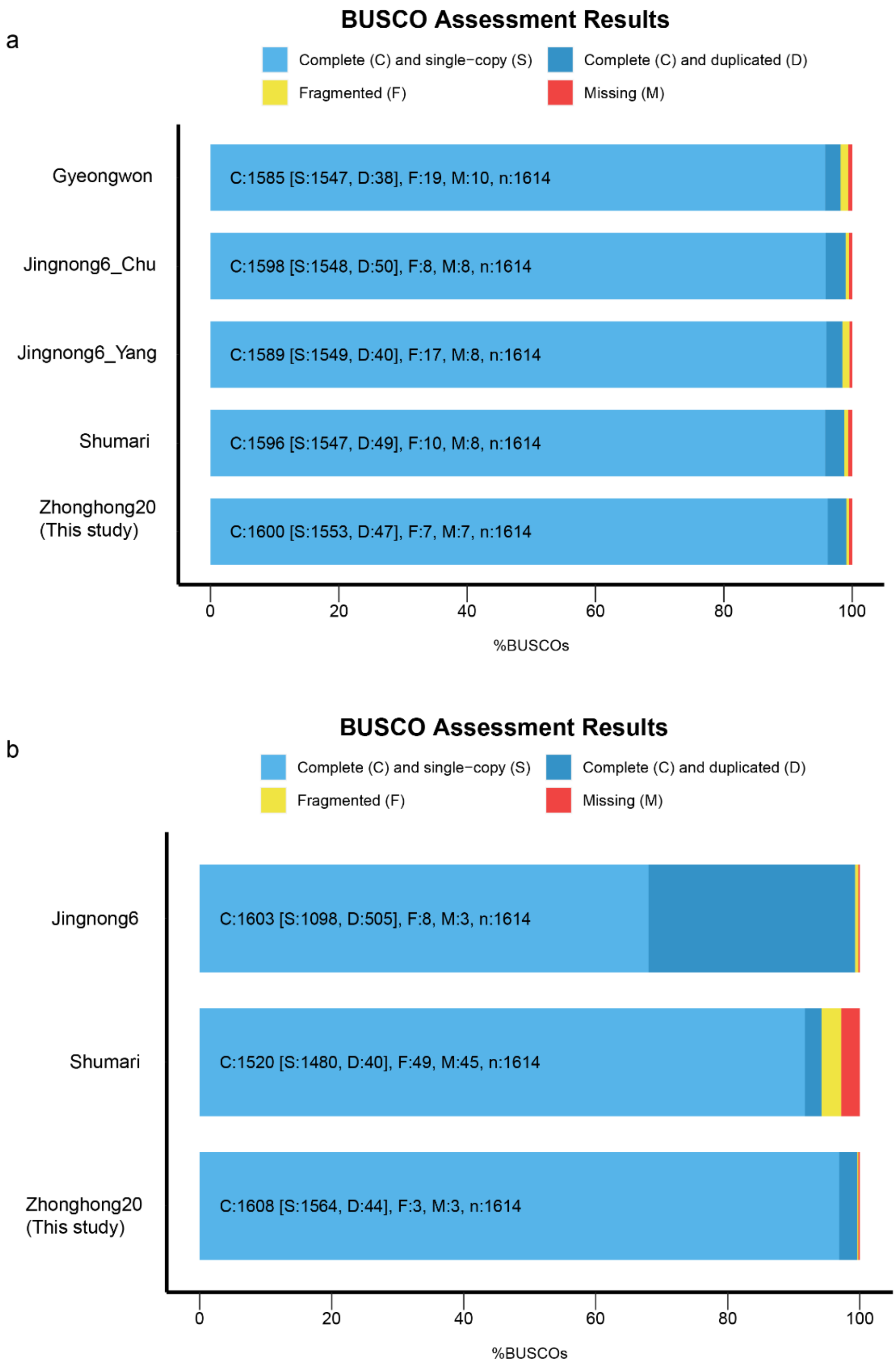

Supplementary Figure 3. Assessment of genome assembly and annotation completeness. Benchmarking Universal Single-Copy Orthologs (BUSCO) analysis against the embryophyta_odb10 database. The results show the completeness scores for **a)** the genome assembly and **b)** the predicted gene set for 'ZH20' and three previously published *Vigna angularis* genomes.

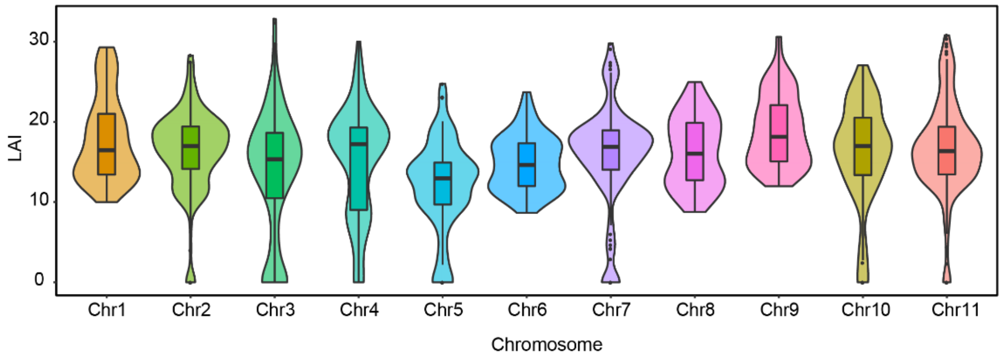

Supplementary Figure 4. LTR Assembly Index (LAI) for the 'ZH20' genome.
The LAI score is shown for each of the 11 pseudochromosomes, indicating the contiguity and quality of the assembly, particularly in repetitive regions.

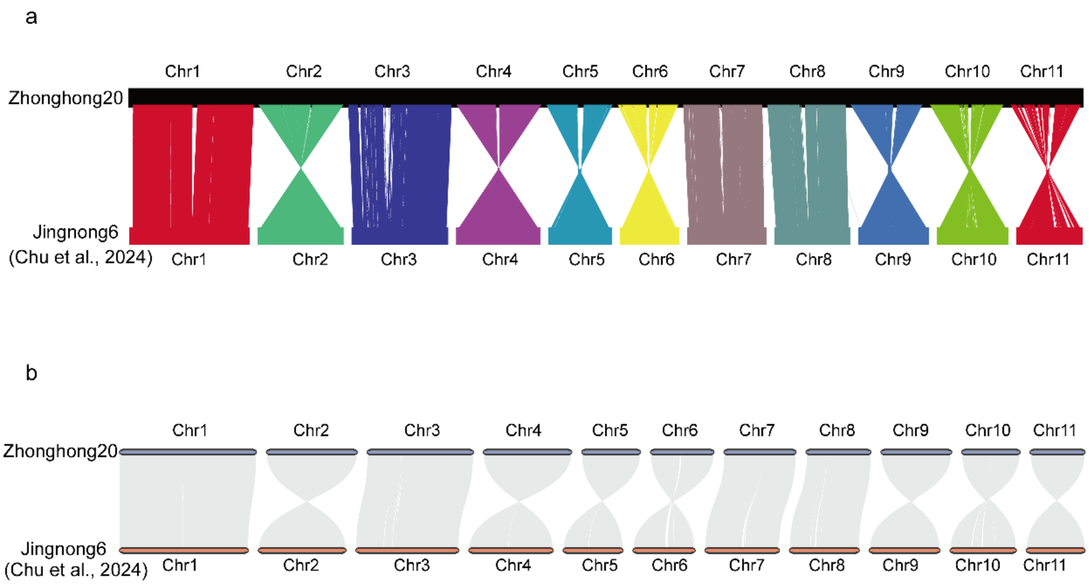

Supplementary Figure 5. Synteny analysis between the 'ZH20' and 'Jingnong6' genomes. **a)** Whole-genome collinearity analysis revealing large-scale syntenic blocks between the two genomes. **b)** Gene-level collinearity analysis highlighting the highly conserved gene order.

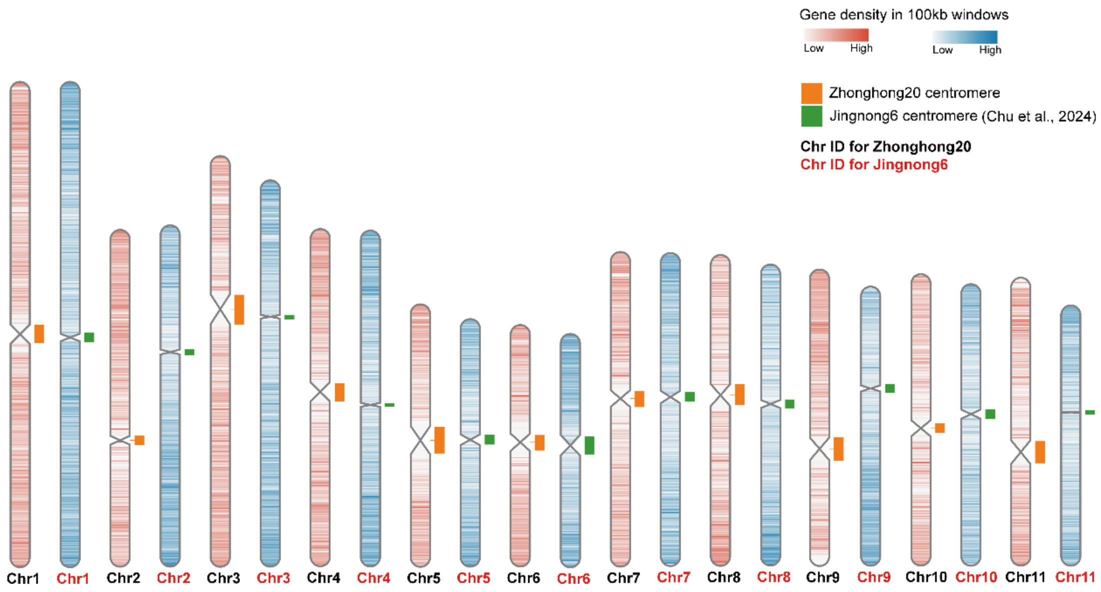

Supplementary Figure 6. Comparison of assembled centromere lengths between 'ZH20' and 'Jingnong6'.

The total length of assembled centromeric sequences for each chromosome is compared for 'ZH20' (red) and 'Jingnong6' (blue), calculated in 0.1 Mb windows.

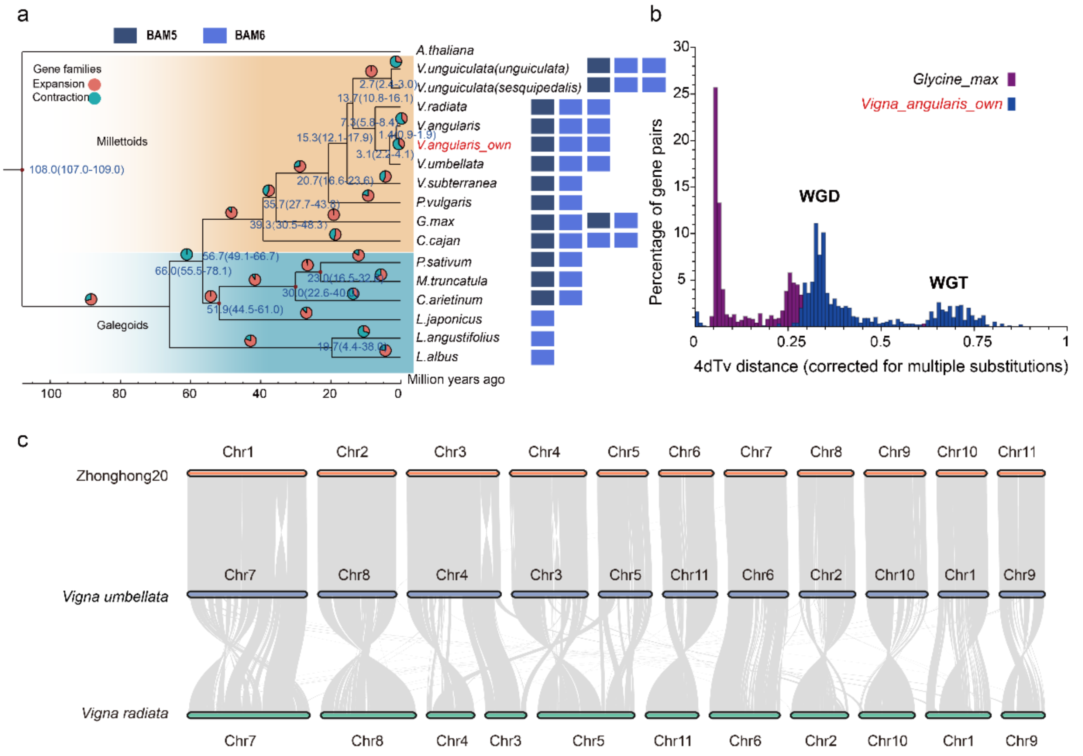

Supplementary Figure 7. Comparative genomic analyses of the 'ZH20' genome.

a) Phylogenetic tree, estimated divergence times, and analysis of gene family expansion and contraction across 15 representative plant species. **b)** Four-fold synonymous third-codon transversion (4DTV) analysis, indicating whole-genome duplication (WGD) events. The peak near 0.3 corresponds to a legume-specific WGD, while the peak near 0.7 corresponds to the gamma whole-genome triplication (WGT) event in eudicots. **c)** Comparative synteny analysis between the genomes of 'ZH20' (*V. angularis*), rice bean (*V. umbellata*), and mung bean (*V. radiata*).

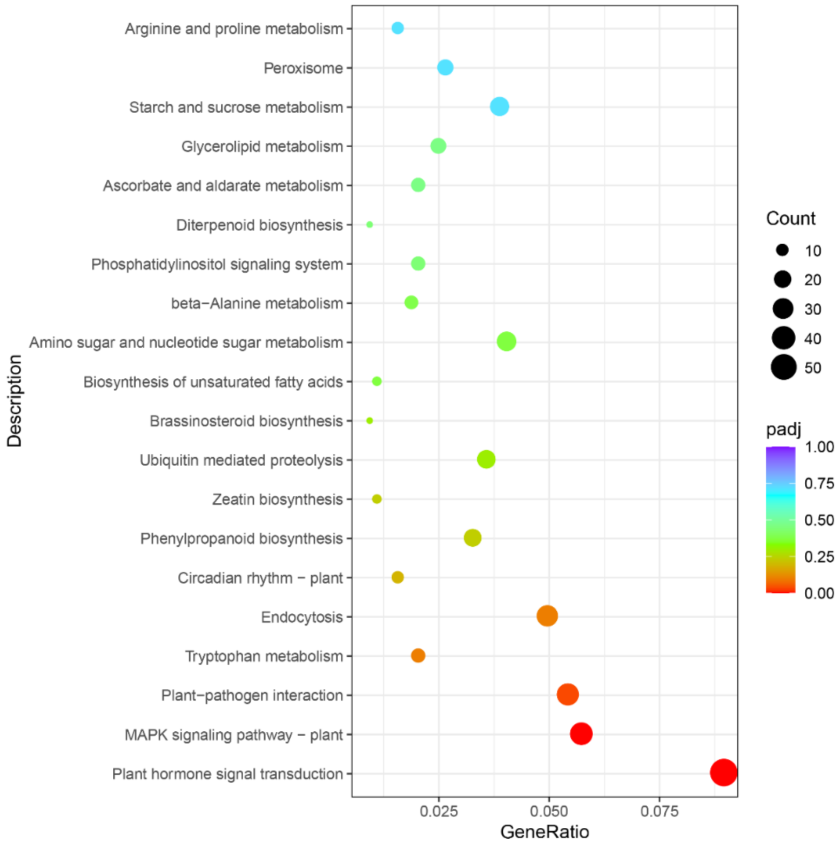

Supplementary Figure 8. KEGG pathway enrichment of genes retained from whole-genome duplication (WGD) events.

The bubble chart shows enriched KEGG pathways. The color of each dot represents the significance of enrichment, while the size corresponds to the number of WGD-derived genes in that pathway.

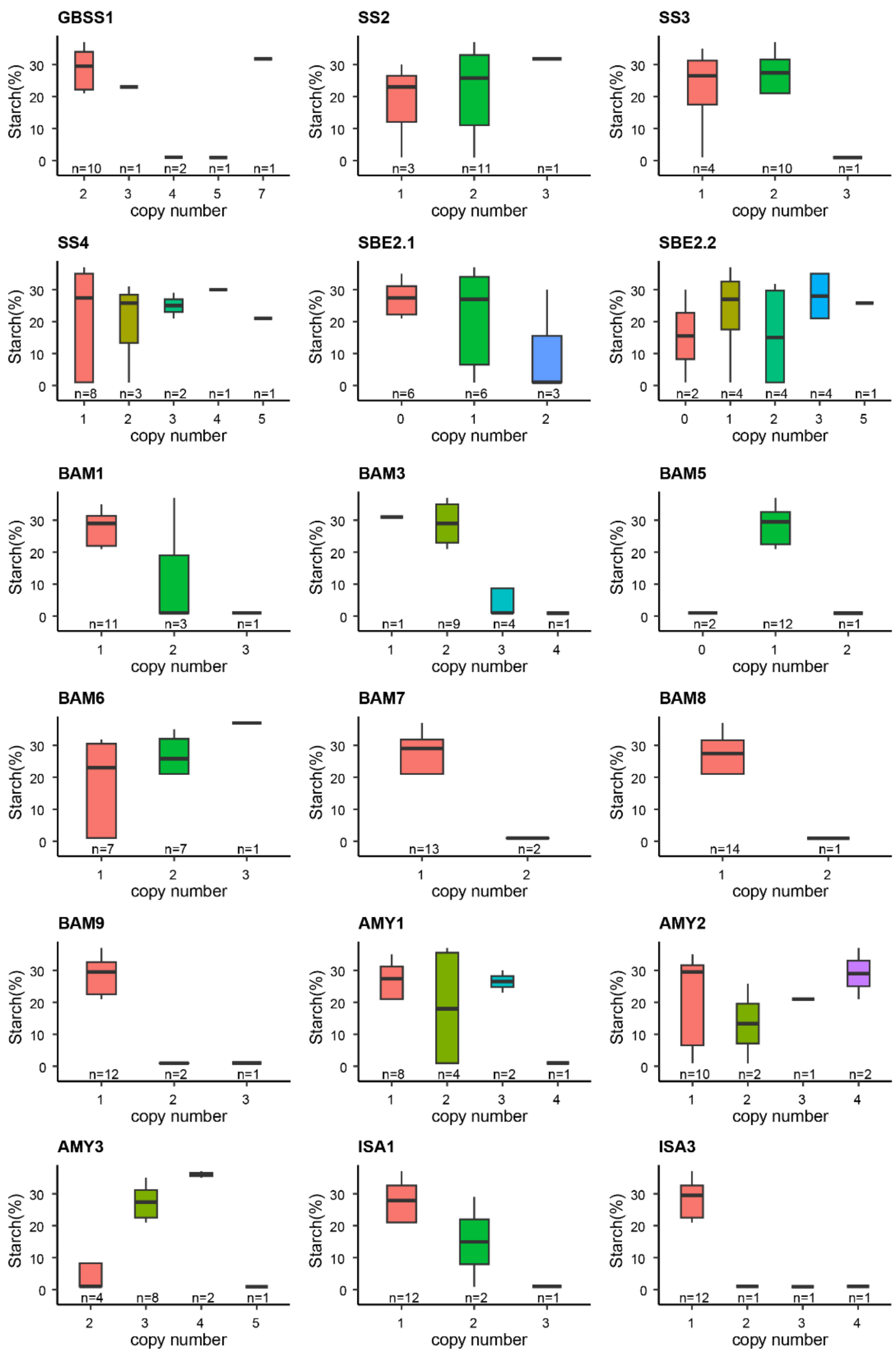

Supplementary Figure 9. Copy number of starch metabolism-related genes across selected legume species.

The heatmap displays the copy number ('n') of key genes involved in starch metabolism, highlighting variations among the compared species.

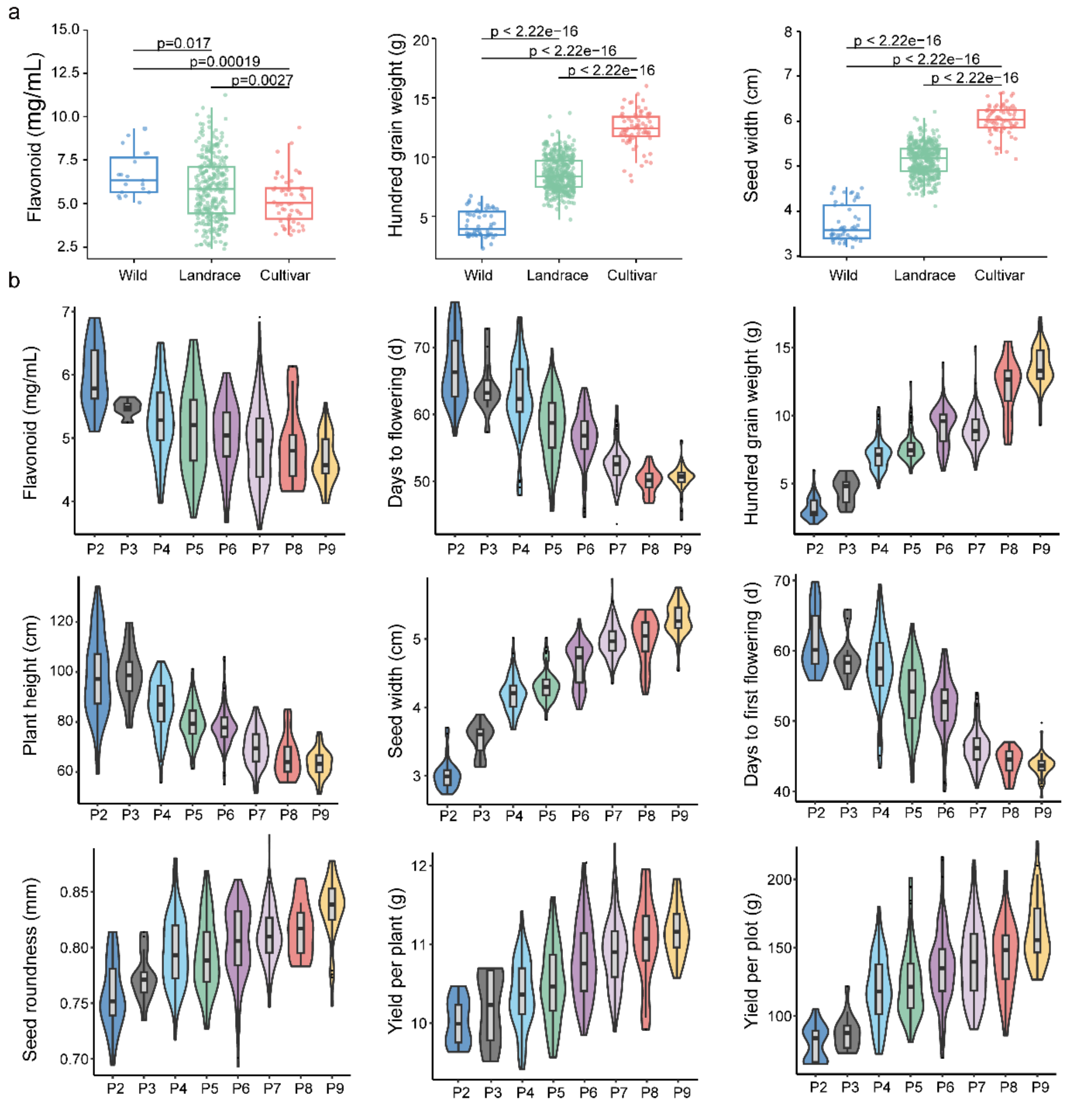

Supplementary Figure 10. Phenotypic diversity and population structure.

a) Distribution of phenotypic values for flavonoid content, hundred-grain weight, and seed diameter across wild, landrace, and cultivated groups. *P*-values were calculated using a two-sided Student's *t*-test. **b)** Distribution of Best Linear Unbiased Estimates (BLUEs) for nine agronomic traits across the eight defined populations (P2–P9).

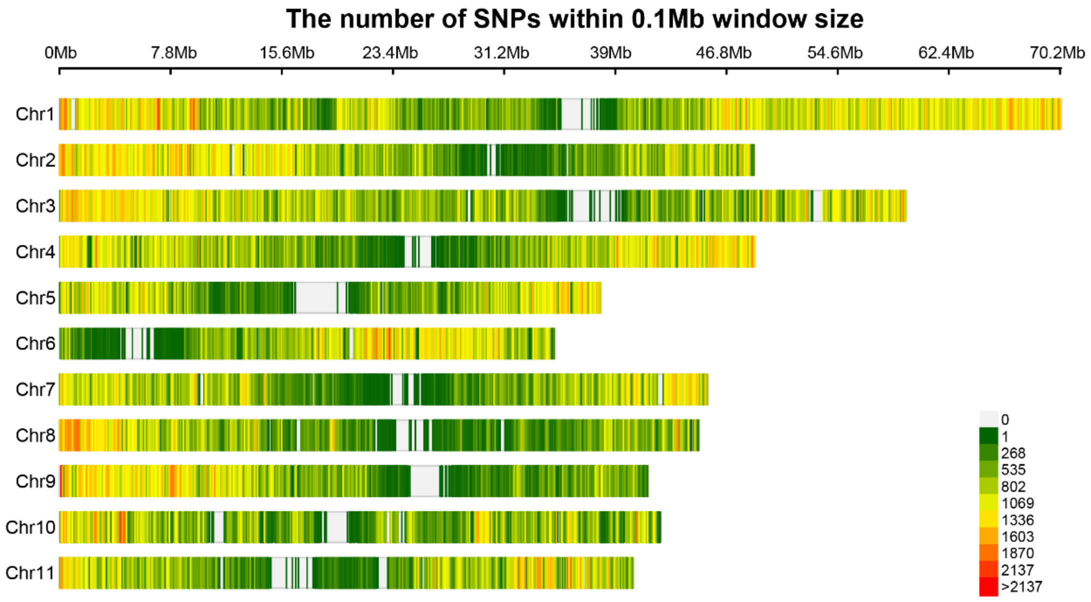

Supplementary Figure 11. Genome-wide SNP density map.

SNP density across the 11 chromosomes, calculated in 0.1 Mb windows. Red indicates high SNP density, green indicates low density, and white indicates no SNPs.

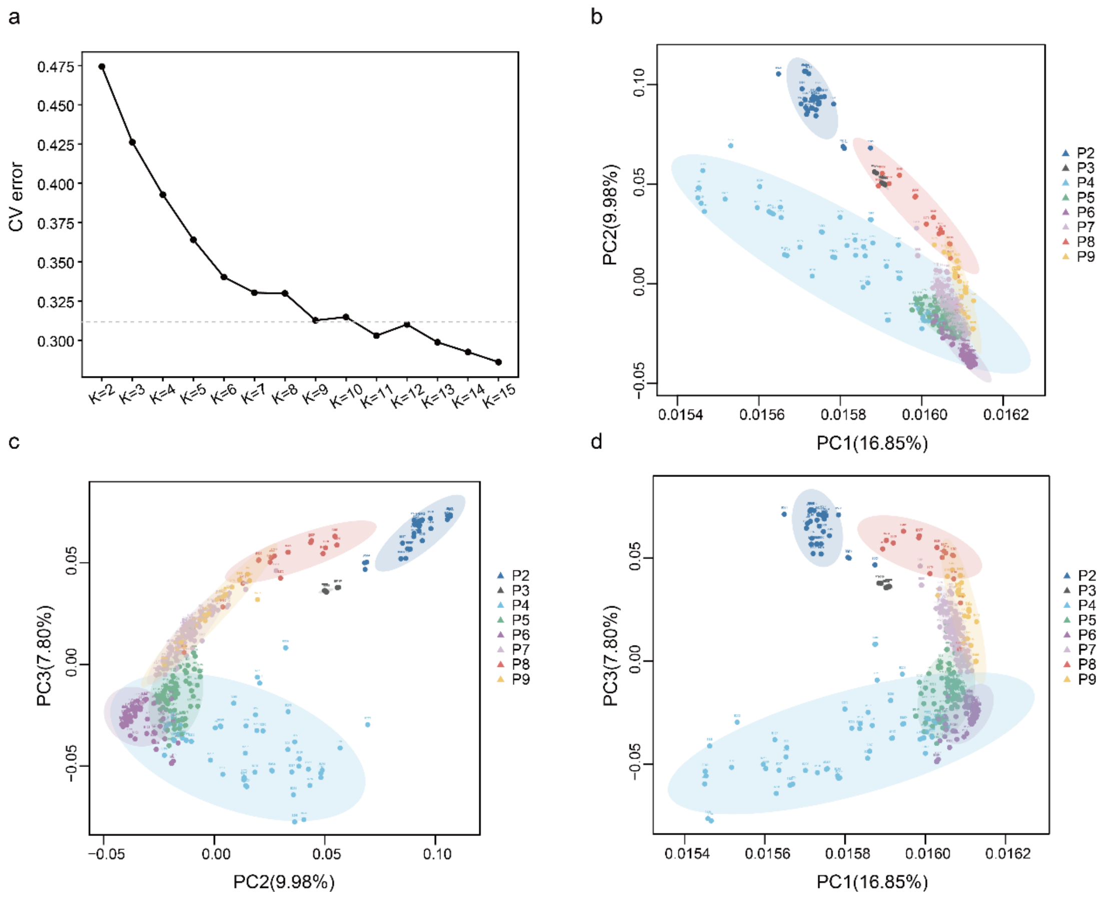

Supplementary Figure 12. Population structure analysis of 546 adzuki bean accessions. **a)** Cross-validation error plot from ADMIXTURE analysis, showing the optimal number of ancestral populations (*K*). **b–d)** Principal Component Analysis (PCA) plots based on genome-wide SNPs, showing the genetic relationships among accessions along **b)** PC1 vs. PC2, **c)** PC1 vs. PC3, and **d)** PC2 vs. PC3.

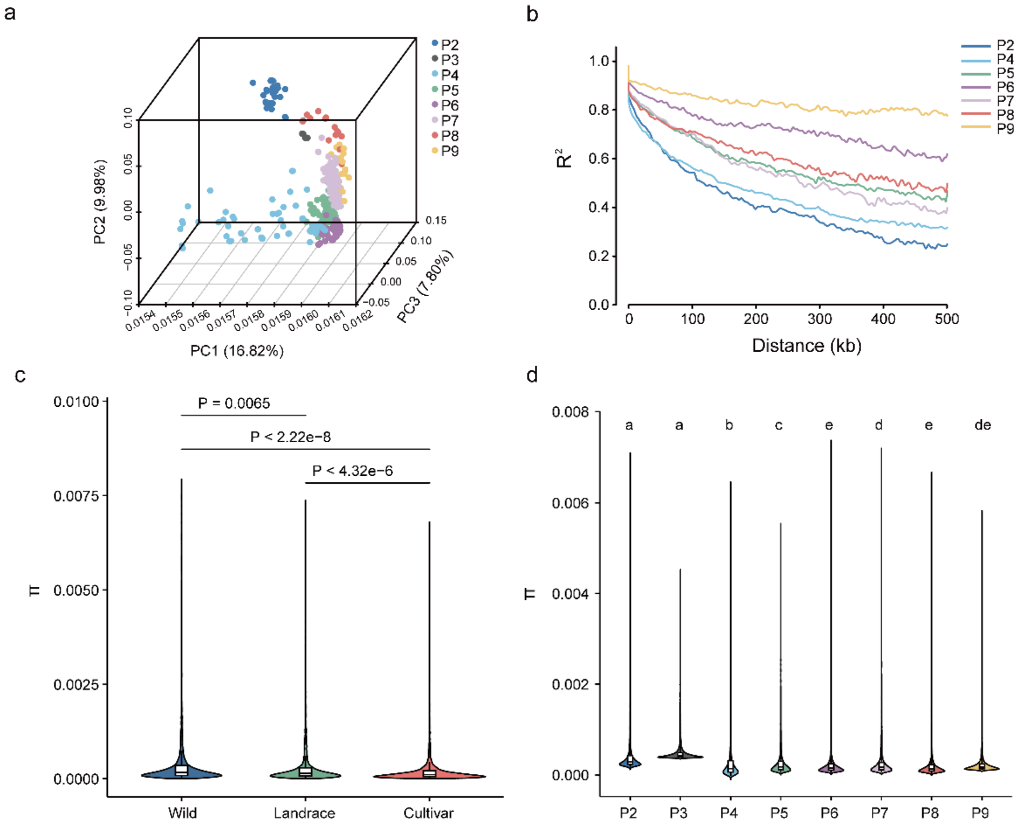

Supplementary Figure 13. Genetic diversity and linkage disequilibrium within populations.

a) Principal Component Analysis (PCA) plot displaying the genetic structure of the 546 accessions. **b)** Genome-wide linkage disequilibrium (LD) decay plots for each of the eight populations (P2–P9). **c)** Comparison of nucleotide diversity (π) among wild, landrace, and cultivated groups. **d)** Nucleotide diversity (π) for each of the eight populations (P2–P9). Significance was assessed using a two-sided Student's *t*-test.

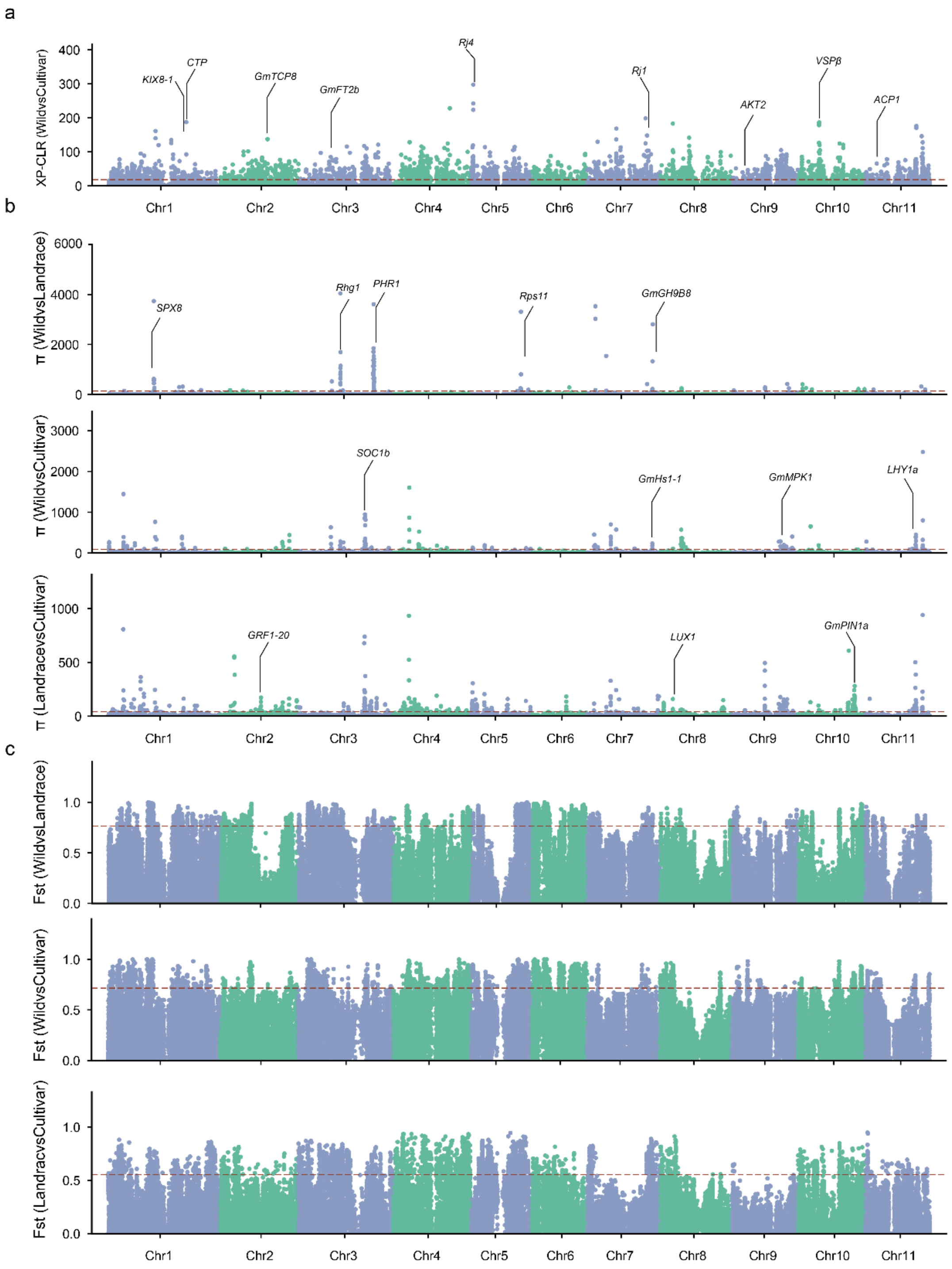

Supplementary Figure 14. Genome-wide scans for selective sweeps.

Manhattan plots showing selection signals identified during adzuki bean domestication and improvement using three different methods. **a)** XP-CLR analysis comparing wild vs. cultivar groups. **b)** Nucleotide diversity ratio (πwild/πlandrace, πwild/πcultivar, πlandrace/πcultivar). **c)** Population fixation statistics (F_{ST}) analysis between population pairs.

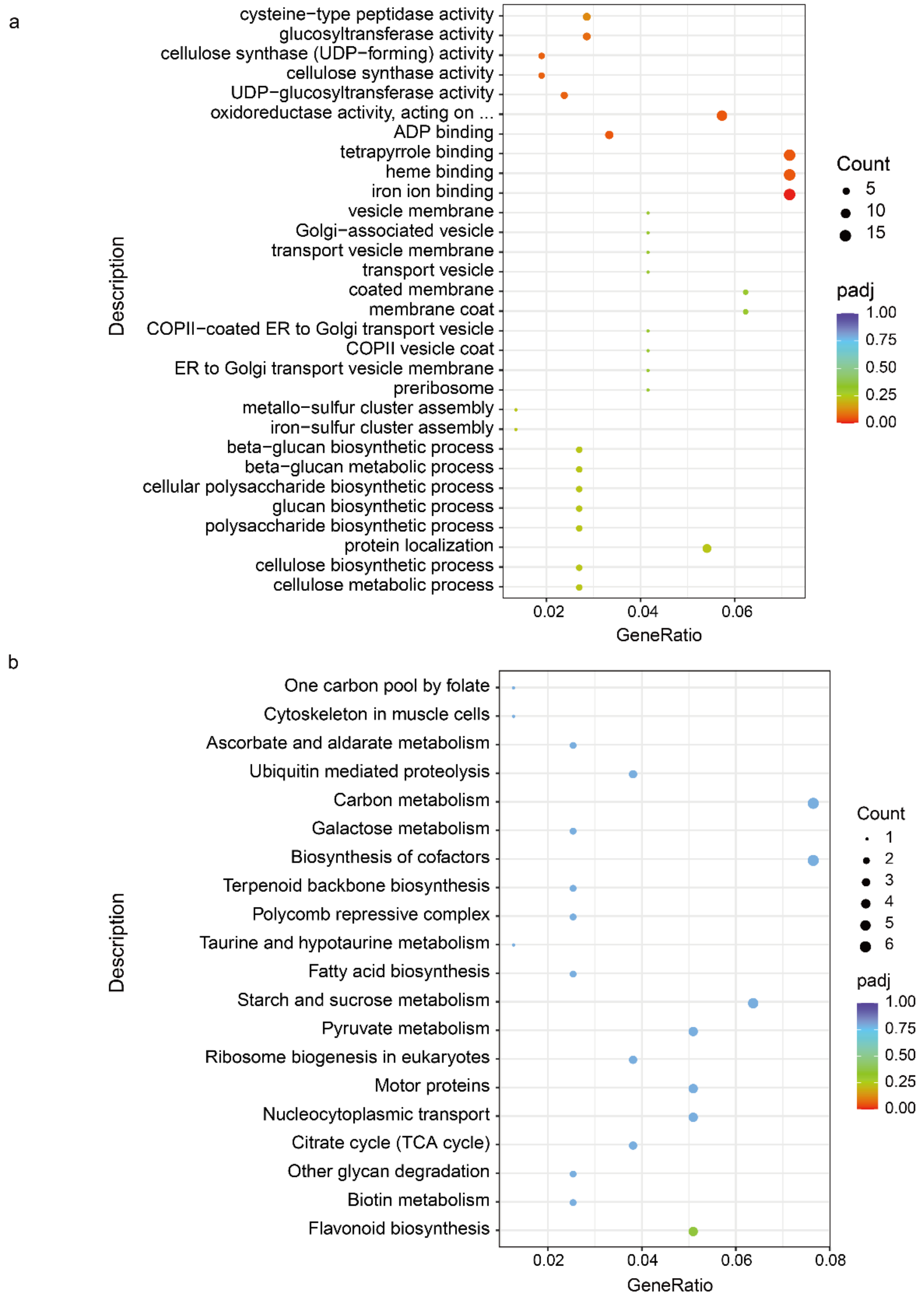

Supplementary Figure 15. Functional enrichment of genes in selective sweep regions.
a) Gene Ontology (GO) term enrichment and **b)** KEGG pathway enrichment for genes located within candidate selective sweep regions. Dot color represents the significance of enrichment, and dot size represents the number of genes.

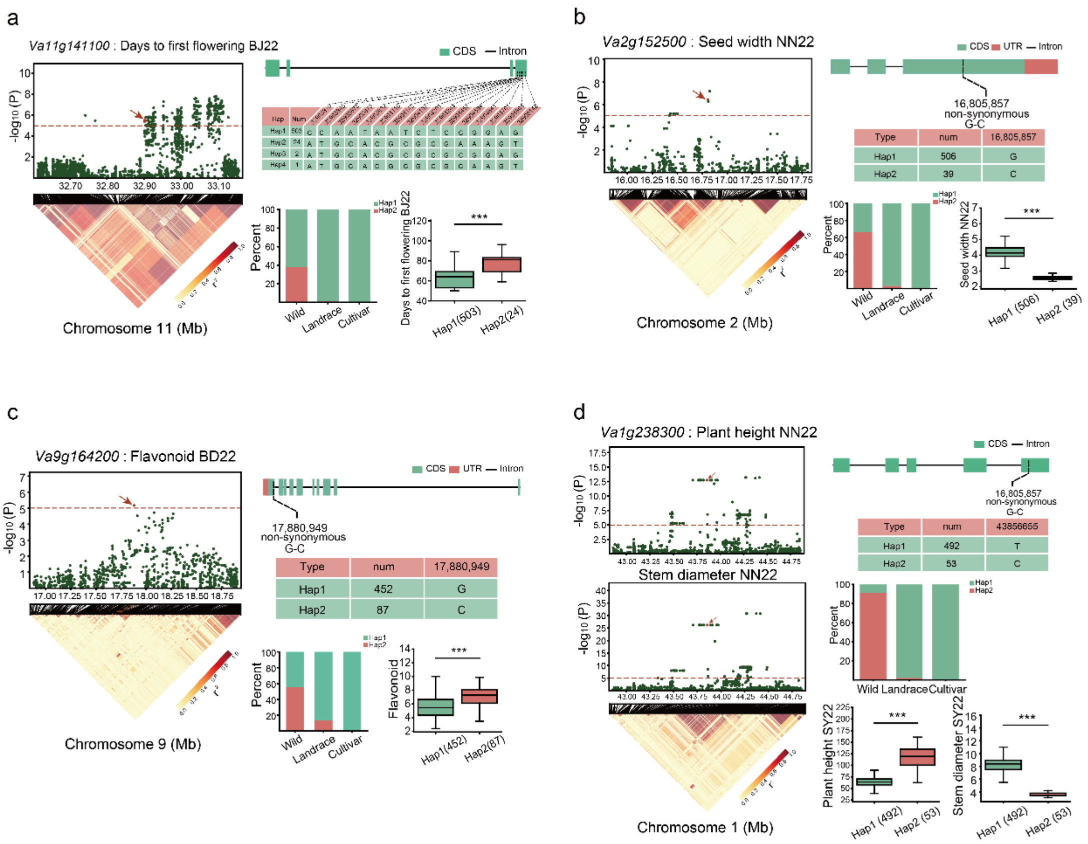

Supplementary Figure 16. Haplotype analysis of candidate genes.

Detailed analysis for four representative candidate genes (**a–d**). For each gene, panels show: the regional GWAS Manhattan plot, gene model, table of haplotypes based on non-synonymous SNPs, haplotype frequency distribution, and boxplots of phenotypic effects for each major haplotype. Statistical significance was determined by a Student's *t*-test ($P < 0.05$, *$P < 0.01$, **$P < 0.001$).

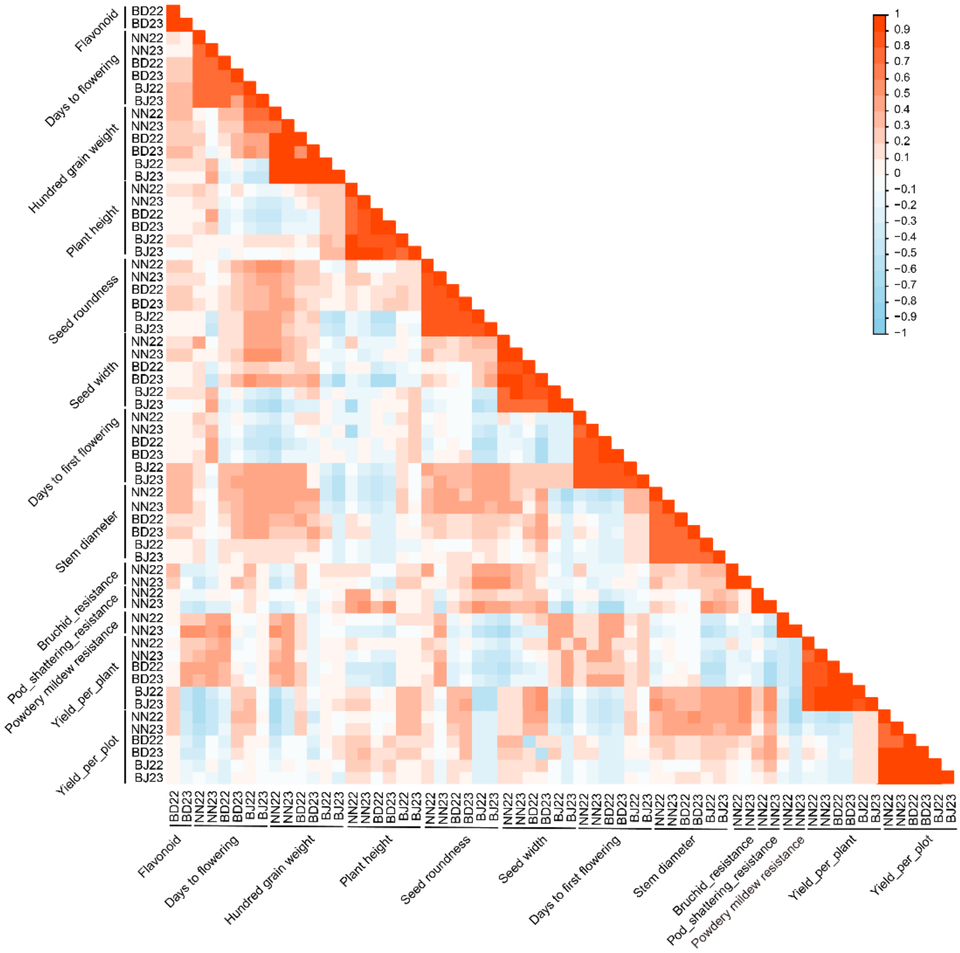

Supplementary Figure 17. Phenotypic correlation matrix.

Pairwise correlations among 13 agronomic traits measured across two growing seasons and three geographical locations.

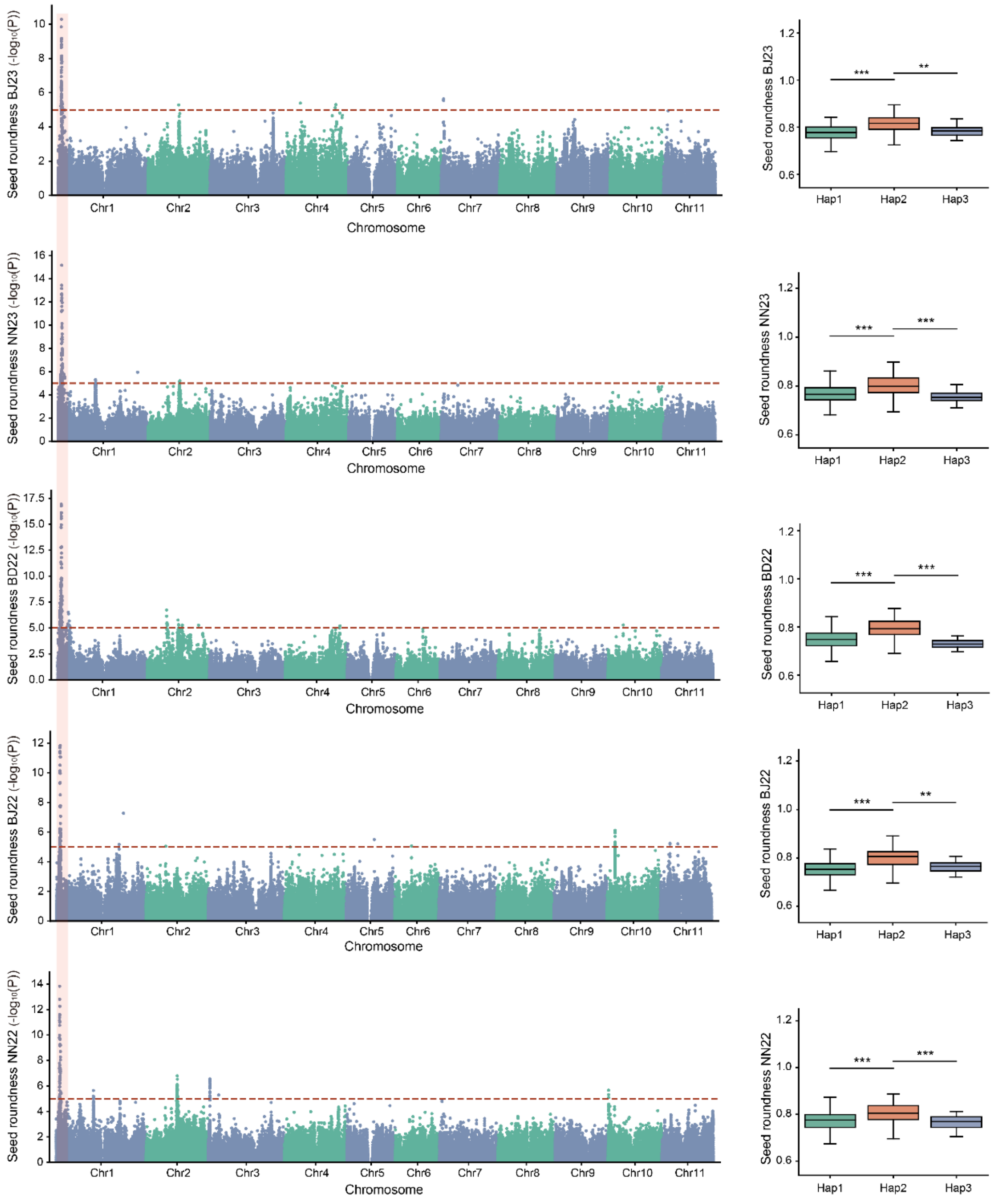

Supplementary Figure 18. GWAS and haplotype analysis for gene *Va1g026200*.
Left: Regional Manhattan plot from the GWAS for seed roundness, centered on the *Va1g026200* locus. Right: Boxplot illustrating the phenotypic effect of different *Va1g026200* haplotypes on seed roundness.

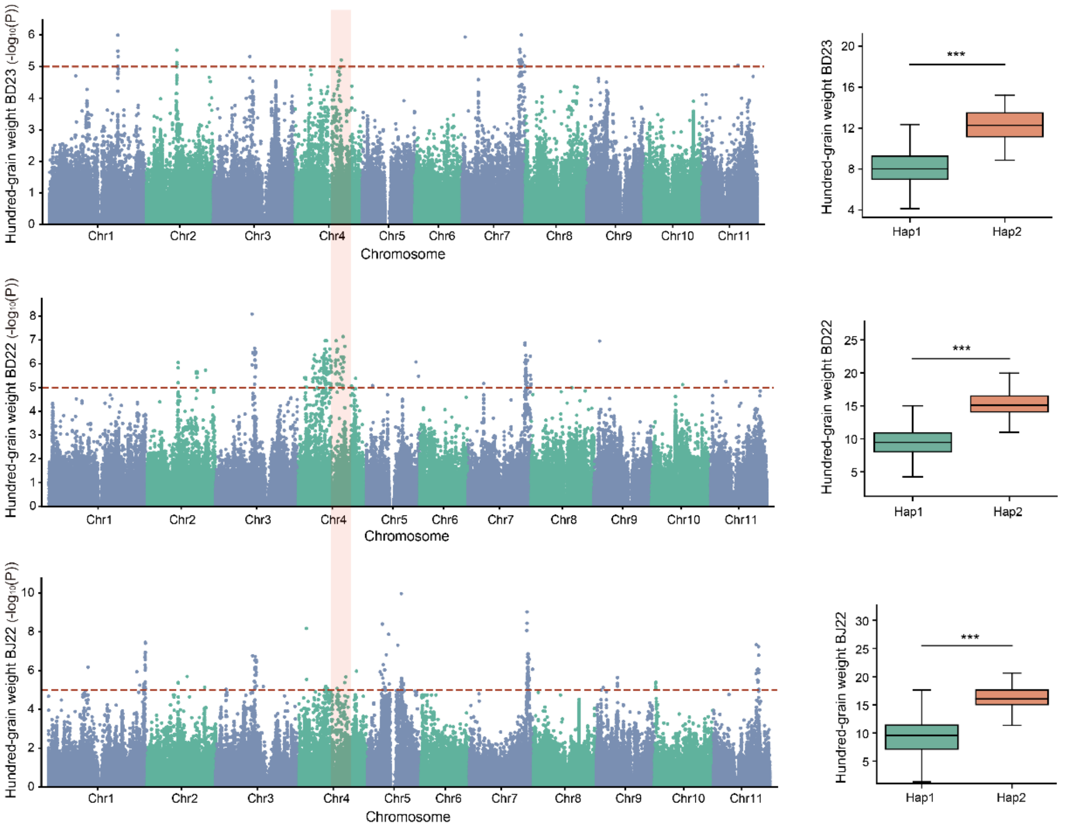

Supplementary Figure 19. GWAS and haplotype analysis for gene *Va4g123700*.

Left: Regional Manhattan plot from the GWAS for hundred-grain weight, centered on the *Va4g123700* locus. Right: Boxplot showing the phenotypic effect of different *Va4g123700* haplotypes on hundred-grain weight.

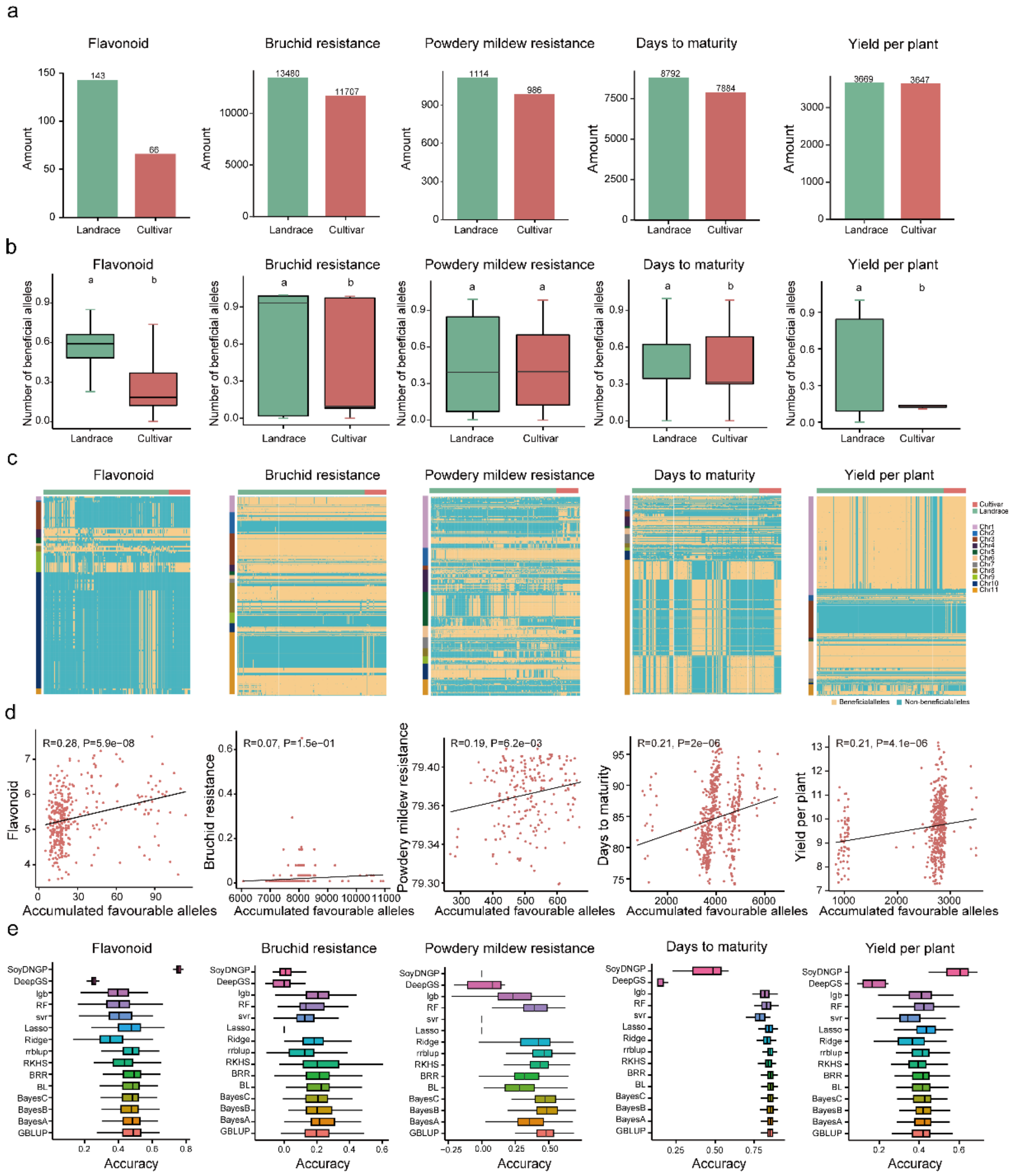

Supplementary Figure 20. Favorable allele analysis and genomic selection.

a) Comparison of favorable allele counts for different traits between landrace (green) and elite cultivar (orange) populations. **b)** Comparison of favorable allele frequencies between populations. Different letters denote significant differences. **c)** Heatmaps showing the distribution of favorable alleles for key traits across all accessions. **d)** Correlation between the cumulative number of favorable alleles and phenotypic values. Pearson correlation coefficient (r) and P-values are shown. **e)** Comparison of genomic prediction accuracy for key traits using 15 different models based on favorable allele loci.
